# TPR is required for the efficient nuclear export of mRNAs and lncRNAs from short and intron-poor genes

**DOI:** 10.1093/nar/gkaa919

**Published:** 2020-10-22

**Authors:** Eliza S Lee, Eric J Wolf, Sean S J Ihn, Harrison W Smith, Andrew Emili, Alexander F Palazzo

**Affiliations:** University of Toronto, Department of Biochemistry, Canada; University of Toronto, Department of Molecular Genetics, Canada; University of Toronto, Department of Biochemistry, Canada; University of Toronto, Department of Biochemistry, Canada; University of Toronto, Department of Molecular Genetics, Canada; Boston University School of Medicine, Department of Biochemistry, Boston, MA, USA; University of Toronto, Department of Biochemistry, Canada

## Abstract

While splicing has been shown to enhance nuclear export, it has remained unclear whether mRNAs generated from intronless genes use specific machinery to promote their export. Here, we investigate the role of the major nuclear pore basket protein, TPR, in regulating mRNA and lncRNA nuclear export in human cells. By sequencing mRNA from the nucleus and cytosol of control and TPR-depleted cells, we provide evidence that TPR is required for the efficient nuclear export of mRNAs and lncRNAs that are generated from short transcripts that tend to have few introns, and we validate this with reporter constructs. Moreover, in TPR-depleted cells reporter mRNAs generated from short transcripts accumulate in nuclear speckles and are bound to Nxf1. These observations suggest that TPR acts downstream of Nxf1 recruitment and may allow mRNAs to leave nuclear speckles and properly dock with the nuclear pore. In summary, our study provides one of the first examples of a factor that is specifically required for the nuclear export of intronless and intron-poor mRNAs and lncRNAs.

## INTRODUCTION

In eukaryotes, mRNA synthesis and processing occurs in the nucleus, while mRNA translation is restricted to the cytoplasm. This subcellular division ensures that only fully processed mRNAs are translated. To accomplish this, mature mRNAs must be recognized and targeted for nuclear export, while pre-mRNAs that have not yet completed splicing must be retained in the nucleus. In addition, cryptic transcripts and misprocessed mRNAs must be nuclear retained and/or degraded.

One of the key components that promote nuclear mRNA export is the Transcription-Export (TREX) complex, which consists of the THOC sub-complex, the mRNA export adaptor Aly (also known as Alyref or REF) and the RNA helicase UAP56 (1–4). This complex is loaded on mRNA and in turn recruits the heterodimeric Nxf1/Nxt1 nuclear transport receptor (also known as TAP/p15) to the mRNA, which helps to ferry it across the nuclear pore (5–9). In addition, a second complex, TREX2, found at the nuclear pore, has also been implicated in mRNA export, although its exact role remains unclear (10–17).

Previously, it was thought that pre-mRNA splicing was a pre-requisite for efficient nuclear mRNA export in metazoans (18,19). Indeed, splicing has been shown to recruit certain TREX components, such as UAP56, to the messenger ribonucleoprotein (mRNP) complex (2,20,21). Nonetheless, TREX components have also been shown to be efficiently loaded onto mRNAs even in the absence of splicing (22–26). TREX may be recruited by RNA polymerase II in a co-transcriptional manner (1), by the nuclear cap-binding complex (27), by exon-splicing elements (28) and by the 3′ cleavage and polyadenylation machinery (29). As such, TREX components are required for the export of non-spliced mRNAs (22–24,28,30). In particular cases where splicing enhances nuclear export, it does so by overriding the activity nuclear retention signals (25), although the exact mechanism for how it does so remains unclear. In addition, it has been reported that additional factors are required for the export of naturally intronless mRNAs (31,32), but to date the role of these factors has not been investigated on a transcriptome-wide scale. Other mRNA features, such as mRNA length may also influence how an mRNA is exported from the nucleus. For example, very short mRNAs (less than 200 nucleotides) do not efficiently recruit the TREX-component Aly and instead are exported by alternative pathways (22,33).

TPR, one of the main components of the nuclear pore basket (34), helps to tether the TREX2 complex to the nucleoplasmic side of the pore (11,14,35). Specifically, TPR interacts with the TREX2 component GANP, which is required for the export of both intronless and spliced mRNAs (14,17,36). Additionally, TPR inhibition by antibody injections, TPR-depletion by RNAi, and overexpression of TPR and TPR fragments, resulted in the nuclear accumulation of poly(A)-RNA – suggesting a role in nuclear mRNA export (11,37,38). Moreover, the yeast homologues of TPR, Mlp1/2, interact with the TREX component protein Aly and the poly(A)-binding protein Nab2 (39–42), which has been implicated in mRNA export in yeast. This interaction may promote the docking of mRNP complexes with the nucleoplasmic face of the yeast nuclear pore (43,44). TREX-Mlp1/2 interactions and mRNP docking could facilitate the exchange of proteins in the mRNP to prepare it for nuclear export (45). Whether TPR associates with TREX components or regulates mRNP remodelling in mammalian cells remains unclear. Since a sizeable fraction of NPC-associated components exist freely in the nucleoplasm, where they are bound to chromatin and regulate gene expression (46–49), it is also possible that soluble TPR may have additional roles in regulating mRNPs even before they reach the nuclear pore.

TPR has also been reported to be required for the nuclear retention of mRNAs with retained introns. In particular, it was seen that TPR-depletion result in an increase of the expression of proteins derived from late HIV transcripts that contain unspliced introns (50,51). In yeast, deletion of the TPR homologues, Mlp1/2, also led to increased protein expression of an intron-containing reporter mRNA, suggesting again that TPR is required for nuclear retention of such transcripts (52–55). Together this data suggested that TPR may be involved in recognizing and retaining improperly processed mRNAs that still contain introns. Importantly, these studies have examined protein expression from reporters and never directly observed whether the cytoplasmic/nuclear distribution of these mRNAs was altered. It is likely that mRNAs with retained introns are targeted for nuclear retention by virtue of the fact that they contain intact 5′ splice site (5′SS) motifs (26,56). Indeed, we previously demonstrated that reporter mRNAs that retain 5′SS motifs in the mature mRNA are nuclear retained and accumulate in nuclear speckles (26). Whether TPR regulates the nuclear retention of these reporters has yet to be directly tested.

Here, we show that the nuclear basket protein TPR does not promote the nuclear retention of 5′SS motif containing RNAs, but instead is required for the efficient nuclear export of mRNAs and lncRNAs that are generated from short transcripts, which typically contain few to no introns. By increasing the length of the transcript (usually by increasing number of introns), their export becomes increasingly independent of TPR. In TPR-depleted cells, nuclear retained mRNAs accumulate in nuclear speckles and are targeted for decay even though they have recruited Nxf1. Our results indicate that TPR acts at a late step in the mRNA export pathway and may facilitate the passage of certain mRNAs out of nuclear speckles and through the nuclear pore.

## MATERIALS AND METHODS

### Plasmids constructs, primers and antibodies

Most *ftz* and *βG* reporter constructs in pcDNA3.0 were described as previously (24–26,30). To generate 5′SS-5′-ftz-Δi reporter plasmid, we inserted the consensus 5′SS motif sequence [AAGGTAAGC] into the 5′ end of our *ftz* reporter using the primer sequences F’ 5′-TCA CAA CAG CCG GGA CAA CAC CCG CTT ACC TTC ATG GTG GCG GCG TCG ACA AG-3′ and R’ 5′-TCA CAA CAG CCG GGA CAA CAC CC-3′. To generate the *βG-ftz-2i* reporter plasmid, we amplified the region spanning *βG* exon 1, 2 and the *ftz* intron within the *βG-ftz-intron-EJ1* reporter plasmid using the primer sequence 1F’ 5′-GGT ACC GCC GCC ACC ATG GAA C-3′ and 1R’ 5′-CCT GAA GTT CTC AGG ATC CAC GTG CAG CTT G-3′. We amplified a similar fragment of the *βG-ftz-intron-EJ2*, except we amplified exons 2, 3 and the *ftz* intron using these primer sequences 2F’ 5′-CTG CTG GTG GTC TAC CCT TGG ACC CAG-3′ and 2R’ 5′-CTC GAG CGG CCG CCA GTG TG-3′. For details on *βG-ftz-intron-EJ1* and *βG-ftz-intron-EJ2*, see reference 25. PCR products from both reactions were run on a gel and gel purified (Geneaid Gel purification kit). Next, the PCR products were combined together in equimolar ratios and allowed to hybridize for 10 PCR cycles before adding primers 1F’ and 2R’ to amplify the entire fragment for 25 cycles. The *βG-ftz-2i* was run on a gel and gel purified as above and inserted into the pJET1.2 vector according to the manufacturer's instructions (Thermoscientific). The *βG-ftz-2i* insert was subcloned into pcDNA3.0 using the HindIII and XhoI as described for *c-ftz-Δi* reporter plasmids.

Antibodies used in this study were a mouse monoclonal TPR-specific antibody (Abcam, ab58344) or a rabbit polyclonal TPR antibody (Abcam, ab70610), Nxf1 (rabbit, 53H8, Santa Cruz, sc32319), UAP56 (rat monoclonal (57)), mAb414 (mouse, Abcam, ab24609), tubulin (rat, YL1/2, Invitrogen, MA1-80017), Aly (rabbit polyclonal (2)), Trap-α (rabbit polyclonal (58)), FLAG (mouse monoclonal, Sigma), histone H3 (rabbit polyclonal, Santa Cruz, sc-10809) and histone H2A.Z (rabbit, Millipore, 07-594). All antibodies were diluted 1:1000 for western blotting and 1:100 to 1:250 for immunofluorescence microscopy.

### Cell culture, DNA transfection experiments and Lentiviral delivered shRNA protein depletion

U2OS and HEK293T cells were grown in DMEM media (Wisent) supplemented with 10% fetal bovine serum (FBS) (Wisent) and 5% penicillin/streptomycin (Wisent). DNA transfection experiments were performed as previously described (26).

The Flp-In T-Rex system (Invitrogen) was used to generate the FLAG-TPR and FLAG-TPR-shR cell U2OS lines as previously described (59). The TPR gene was subcloned from FLAG-TPR pcDNA3.0 (Addgene plasmid # 60882) (60) to pcDNA5.0. To generate a shRNA resistant *FLAG-TPR* gene (*FLAG-TPR-shR*), we made synonymous mutations in the sequence targeted by shRNA TPR A (nucleotide 2480–2501) using the primer sequence F’ 5′-TCTGCAAACA ATTCAGGGAA TACTGGAAAG GAGTGAGACT GAGACGAA-3′ and R’ 5′-ACAAAGGCTT AGTAGCCAGA TAGAAAAACT G-3′. The Flp-In U2OS cell lines were generated according to manufacturer's instructions and selected using 5 μg/ml blasticidin and 200 μg/ml hygromycin for 3 weeks until individual colonies can be isolated. To induce the expression of FLAG-TPR and FLAG-TPR-shR, cells were treated for 48 h of 2 μg/ml doxycycline, and fixed and stained as described below.

The lentiviral delivered shRNA protein depletion was performed as previously described (24,25,61). Briefly, HEK293T were plated at 50% confluency on 60 mm dishes and transiently transfected with the gene specific shRNA pLKO.1 plasmid (Sigma), packaging plasmid (Δ8.9) and envelope (VSVG) vectors using Lipo293T DNA *in vitro* transfection reagent (SignaGen Laboratories) according to the manufacture's protocol. 48 h post-transfection, viruses were harvested from the media and added to U2OS cells pre-treated with 8 μg/ml hexadimethrine bromide. Cells were selected with 2 μg/ml puromycin media for at least 4 to 6 days. Western blotting was used to determine the efficiency of TPR depletion. The shRNAs constructs (Sigma) used in this study are as follows: ‘TPR A’ shRNA, TRCN0000060066 (5′-CCGGGCGATC TGAAACAGAA ACCAACTCGA GTTGGTTTCT GTTTCAGATC GCTTTTTG-3′) and ‘TPR D’ shRNA, TRCN0000060067 (5′-CCGGCGTAGG TACAAGACTC AATATCTCGA GATATTGAGT CTTGTACCTA CGTTTTTG-3′).

### Microinjection, fluorescent in situ hybridization (FISH) staining, immunostaining and nuclear speckle Pearson correlation and enrichment quantifications

The DNA and RNA microinjection experiments, cell fixation, FISH and immunofluorescence staining, imaging and mRNA distribution analysis were performed as previously described (24–26,30,62,63). Briefly, for DNA injections, pcDNA3.0 plasmid containing the *ftz-Δi* reporter gene (26) was microinjected at 400 ng/μl with 70kDa Oregon Green Dextran (ThermoFisher). After allowing RNA synthesis to occur for 20 min at 37°C, further transcription was inhibited with 1 μg/ml α-amanitin and cells were incubated at 37°C for the indicated times. Cells were then fixed (4% paraformaldehyde in PBS), stained, and imaged. *MHC-ftz-Δi* mRNA was synthesized *in vitro* using HiScribe T7 High Yield RNA Synthesis Kit (New England Biolabs) with *MHC-ftz-Δi* in pcDNA3.0 (30) digested with XhoI as a template. The RNA was capped with Vaccinia Capping System (New England Biolabs) and polyadenylated with E. coli Poly(A) Polymerase (New England Biolabs) following the manufacturers’ protocol. RNA was microinjected at 600 ng/μl with 70 kDa Oregon Green Dextran. Injected cells were incubated at 37°C for the indicated times then fixed (4% paraformaldehyde in PBS), stained, and imaged. Alexa546-conjugated FISH probes and the staining procedure were described previously (24,30). SC35 immunostaining using mouse monoclonal anti-SC35 (Clone SC35, Sigma; 1:1500–1:2000 dilution) and secondary antibody conjugated to Alexa 647 (Molecular Probes) was performed as described previously (24).

**Figure 1.**
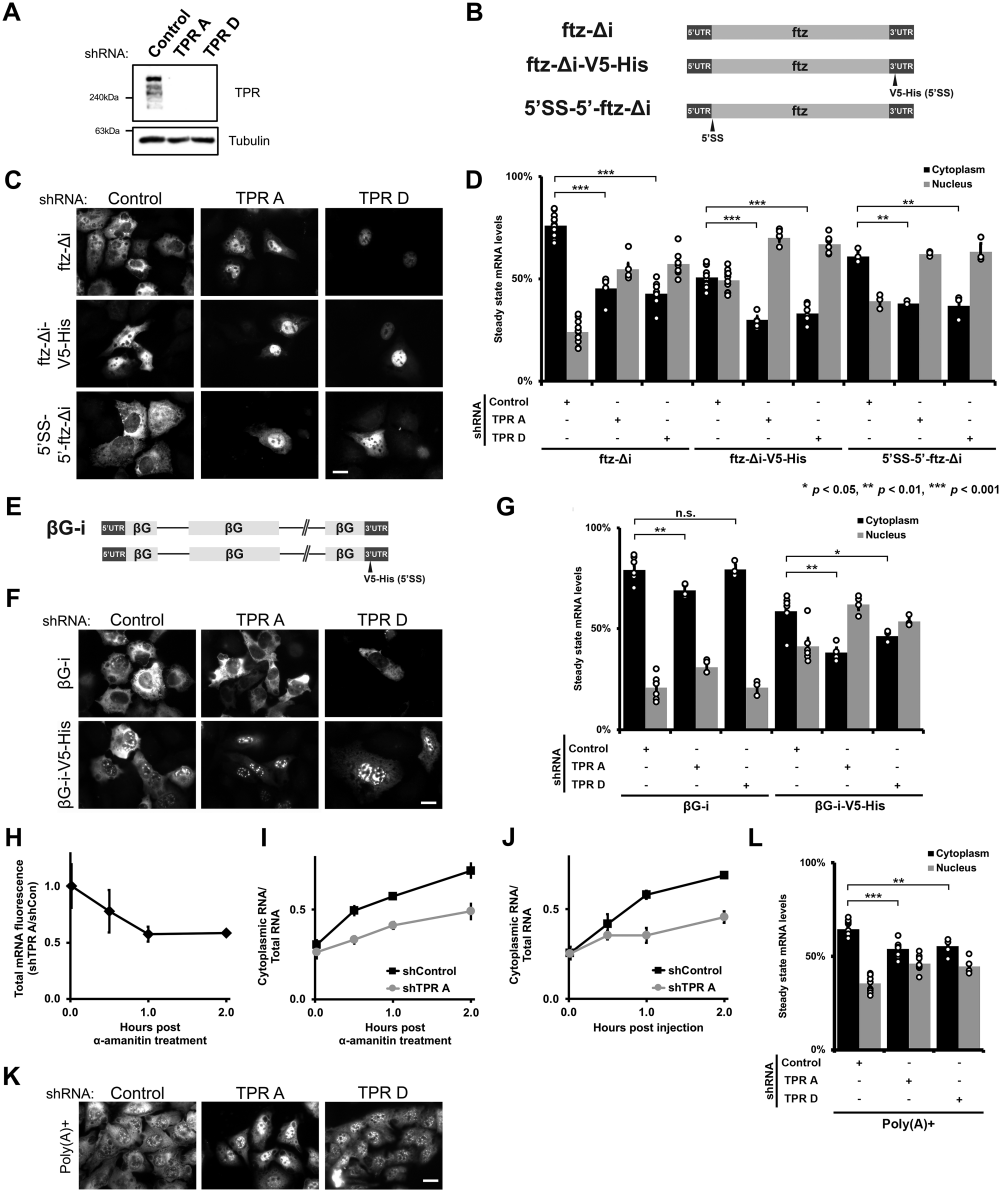
TPR is required for the cytoplasmic accumulation of certain reporter mRNAs but not the nuclear retention of 5′SS motif containing mRNAs. (**A**) U2OS cells were treated with two different lentiviral delivered shRNAs against TPR (‘TPR A’ and ‘TPR D’) or scrambled control. Lysates were collected after 96 h, separated by SDS-PAGE and immunoprobed for TPR or tubulin. (**B**) Schematic of the intronless *ftz* reporter construct used in this study, with and without the *V5-His* element in the 3′ UTR. The *V5-His* element contains the 5′SS motif which promotes nuclear retention. For *5*′*SS-5*′*-ftz-Δi*, the 5′SS motif was inserted into the 5′ end of the *ftz* reporter. (**C, D**) Control- or TPR-depleted cells were transfected with the intronless *ftz* reporter plasmid (±*V5-His*). 18–24 h later the cells were fixed and the mRNA was visualized by FISH. TPR depletion caused nuclear accumulation of the *ftz-Δi* mRNA, irrespective of the 5′SS motif. Representative images are shown in (**C**), scale bar = 10 μm, and quantification is shown in (**D**) with each bar representing the average and standard error of at least three independent experiments, each experiment consisting of at least 30 to 60 cells. Student t-test was performed for Figure 1D, G and L, * *P* < 0.05, ** *P* < 0.01, *** *P* < 0.001. (**E**) Schematic of the intron containing *βG-i* reporter mRNA, with and without the *V5-His* element. (**F, G**) Similar to (C, D), except that *βG-i* reporter plasmid was used. TPR depletion did not significantly affect the mRNA distribution of the *βG-i* reporter mRNA but increased the nuclear accumulation of *βG-i-V5-His* mRNA. Scale bar = 10 μm. Representative images are shown in (**F**), and quantification is shown in (**G**) with each bar representing the average and standard error of at least three independent experiments, each experiment consisting of at least 30 to 60 cells. (H, I) *ftz-Δi* plasmid was microinjected into the nuclei of Control- or TPR-depleted U2OS cells. After allowing mRNA synthesis for 20 min, cells were treated with α-amanitin and then incubated for various times to allowed for mRNA export. Cells were fixed, and mRNA was visualized by FISH. Each point is the average and standard error of at least five independent experiments, each of which consist of 30–45 cells. (**H**) To determine whether TPR-depletion promotes the degradation of newly synthesized mRNAs, the ratio of *ftz-Δi* signal in the control and TPR-depleted cells were plotted over time. Note that the relative level of *ftz-Δi* in TPR-depleted cells decreases over the first hour until about 60% of the mRNA remains, after which point the ratio is stable. (**I**) Cytoplasmic/total RNA ratio was quantified in control and TPR-depleted cells. (**J**) Cells were microinjected with *in vitro* synthesized *MHC-ftz-Δi* RNA, which was capped and polyadenylated, along with 70kDa Oregon Green Dextran to mark the injected compartment. The microinjected control- or TPR-depleted U2OS cells were fixed after various time points post injection. mRNA export was monitored in nuclear injected cells by FISH and the cytoplasmic/total distribution was quantified as in (I). Each point is the average and standard error of at least four independent experiments, each of which consists of 20–40 cells. (**K, L**) TPR depletion causes nuclear accumulation of poly(A)+ RNAs, as visualized by oligo-dT FISH staining, scale bar = 10 μm. Representative images are shown in (**K**), and quantification is shown in (**L**) with each bar representing the average and standard error of at least eight independent experiments for Control- or ‘TPR A’ depleted cells and four independent experiments for ‘TPR D’ depleted cells, each experiment consisting of at least 30 cells.

The Pearson correlation quantification of mRNA in speckles was performed as previously described (24). Note that this quantification method has been validated with extensive controls—see reference 24, Figure 1D–G. For each experiment, the 10 brightest SC35-positive speckles per cell were analyzed and the totals from 15 to 20 cells were averaged. This analysis was repeated three times and the averages and standard errors between experiments were determined and graphed.

The nuclear speckle enrichment of *ftz* mRNA was performed as previously described (24). In brief, thresholds were drawn using the SC35 immunofluorescence channel and set so that 10% (±0.5%) of the nuclear area was selected per cell. The fluorescence intensity of RNA in the selected area, in the nucleus and in the entire cell were calculated.

### Stellaris single molecule (sm)FISH experiments

Stellaris smFISH experiments were performed as previously described (64) and following the manufacturer's protocol with a few modifications. Following fixation with 4% paraformaldehyde (Electron Microscopy Sciences), coverslips were methanol treated for 30 min in −20°C and rehydrated twice in 1X PBS, each time washing for 5 min. Cells were washed twice in 2× SSC buffer (150 mM NaCl, 15 mM NaCitrate, pH 7.1) with 10% formamide. 100 μl of hybridization probe (100 mg/ml dextran sulfate, 10–15% formamide, 2× SSC) containing 125 nM Stellaris probes (LGC Biosearch Technologies) was added to the coverslips and incubated for 48 h. Subsequently, the coverslips were washed three times with 2× SSC buffer with 10–15% formamide and mounted onto coverslips using DAPI Fluoromount-G stain mounting solution (Southern Biotech). Cells were imaged and quantified as previously described (64).

### RNA-IP experiments

The Nxf1/TAP RNA-IP experiments were performed exactly as previously described (25,26), except that TPR-depleted cells were transfected in normal DMEM media without puromycin. For the TPR-FLAG IP experiments, FLAG-TPR Flp-In U2OS cells were treated with 1 μg/ul doxycycline for 4 h, media was removed and subsequently co-transfected with reporter plasmids. Cells were harvested 18–24 h post-transfection and the RNA-IP experiments was performed using the same conditions as for the Nxf1/TAP RNA-IP. Superscript IV reverse transcriptase (Invitrogen) was used for Nxf1/TAP RNA-IP and TPR-FLAG IP. To access the efficiency of the TPR-FLAG IP, samples were separated on an SDS-PAGE gel and transferred onto a blot for immunoblotting.

### RNA Frac-Seq

2 × 150 mm dishes of U2OS cells at 60–80% confluency cells were depleted of TPR using shRNA ‘TPR A’ or ‘TPR D’ for 5 days. Cells were isolated by centrifugation at 800 *g*, washed with 1× PBS 3 times and 10% of cell volume was reserved as the ‘total RNA’ fraction. The remaining 90% was resuspended in 500 μl φ buffer [150 mM potassium acetate, 5 mM magnesium acetate, 20 mM HEPES pH 7.4, 1 mM sodium fluoride, 1 mM sodium orthovanadate, 25× protease inhibitor cocktail (Roche), 1:1000 dilution of SUPERase In™ RNase (Invitrogen) and 0.1% diethylpyrocarbonate]. 500 μl of φ buffer with 1% Triton X-100 (Thermoscientific) and 0.2% sodium deoxycholate was gently added to the resuspended cells and incubated on ice for 3 min. The mixture was centrifuged at 800 *g* for 5 min and the supernatant (the ‘cytoplasmic/ER fraction’) was removed and respun at 16 100 *g* for 5 min to clear any cell debris. The resulting supernatant was transferred to a new 15 ml falcon tube and 2ml of TRIZOL (Life Technologies) was immediately added. The cell pellet (the ‘nuclear fraction’) was resuspended with 500 μl of φ buffer, and 500 μl of φ buffer with detergents was added as before and spun at 800 *g* for 5 min. The supernatant was removed, the cell pellet was washed with 1 ml of φ buffer and was resuspended in 1 ml of φ buffer. Next, 2 ml of TRIZOL was added to the nuclear fraction, 400 μl of chloroform was added and the tube was thoroughly mixed and centrifuged at 12 500 *g* for 15 min. The top layer containing the RNA was removed, washed with chloroform and the RNA was next purified and washed according to manufacturer's instruction for the RNA PureLink column (ThermoFisher Scientific) column. The same RNA extraction procedure was repeated to extract RNA from the ‘total’ fraction. To monitor the purity of the cell lysate fractions, 50 μl of ‘cytoplasmic/ER’ and ‘nuclear’ fractions was denatured with 1X Laemmli Sample Buffer, separated by SDS-PAGE and probed for various compartment specific markers; Aly as the nuclear marker, Trap-α as the ER marker and tubulin as a cytosolic marker.

To prepare the sample for sequencing, 1 μg of DNase I-treated RNA (Ambion DNA-free DNA removal kit (Invitrogen)) was used. To prepare the RNA library, we followed the manufacturer's protocol for the NEBNext^®^ Ultra™ II Directional RNA Library Prep kit (NEB, E7765S) and poly(A) selected the RNA using the NEBNext Poly(A) mRNA Magnetic Isolation Module (NEB, E7490S). We used the NEBNext Multiplex Oligos for Illumina (Index Primers #1–#24) (NEB, E7335S and E7500S) as the unique index primers. RNA quality control was performed at all steps using BioAnalyzer and sequenced at the Donnelly Centre Sequencing facility at the University of Toronto using the Illumnia Hi-Seq500 platform.

### RNA Frac-Seq data processing and analysis

A Salmon index (65) was built from Gencode v.19 protein-coding and long non-coding transcript sequences (66). Fastq files corresponding to each biological replicate were pseudo-aligned to the Salmon index (command: quant –validateMappings). Gene abundances were calculated utilizing Tximport (67). Genes with a mean FPKM > 1 were considered for further analysis and genes from the mitochondrial genome were excluded. Gene-level fold changes in the nuclear to cytoplasmic ratio of TPR and control knockdowns were calculated utilizing DESeq2 (68) (with fold change shrinkage) and compared (formula: log_2_(Nuclear/Cytoplasmic)_TPR(A/D)_ – log_2_(Nuclear/Cytoplasmic)_Control_). Gene-level fold changes in nuclear proportions (formula: log_2_(Nuclear/Total)_TPR(A/D)_ - log_2_(Nuclear/Total)_Control_) and cytoplasmic proportions (formula: log_2_(Cytoplasmic/Total)_TPR(A/D)_ – log_2_(Cytoplasmic/Total)_CTRL_) between TPR and control knockdowns were calculated similarly utilizing DESeq2 (with fold change shrinkage) (68). TPR normalized counts for each biological replicate were calculated utilizing DESeq2 (plotCounts) (68).

Gene length, transcript length (exons-only), total number of exons per transcript, fraction intronic sequence (intronic sequence length per gene/gene length) and exon density (total number of exons per transcript/transcript length) of each transcript were calculated from Gencode v.19 annotation (66) for protein-coding and long non-coding RNA transcripts. A gene-level median of each metric was calculated.

For correlation analysis, spearman correlations of the gene-level median of each metric compared to the fold changes in the nuclear to cytoplasmic ratio between TPR and control knockdowns were calculated.

For length bin analysis, protein coding or long non-coding RNA encoding genes were divided into five bins based upon median pre-mRNA length (<10, 10–20, 20–30, 30–40, 40–50, >50 kb). Genes within each length bin were compared to fold changes in the nuclear to cytoplasmic ratio between TPR and control knockdowns.

For analysis of the total number of exons, multi-exonic genes were defined as genes with a median total number of exons greater than one and single exon genes were defined as genes with a median total number of exons exactly equal to one (unrounded). Multi-exonic and single exon genes were compared to fold changes in the nuclear to cytoplasmic ratio between TPR and control knockdowns. Analysis of genes in particular bins by the total number of exons was performed by rounding the median total number of exons per gene to the nearest integer, followed by comparison to fold changes in the nuclear to cytoplasmic ratio between TPR and control knockdowns. Histone and ribosomal gene annotation were derived from HGNC (69). Statistical analyses were performed utilizing Mann–Whitney–Wilcoxon tests with Bejamini–Hochberg adjustment for multiple comparisons.

## RESULTS

### TPR is not required for the nuclear retention of 5′SS motif containing mRNAs, but is required for the cytoplasmic accumulation of certain reporter mRNAs

Previously, we identified a *cis*-acting RNA element, the 5′SS motif, that inhibits mRNA nuclear export (26). Since TPR has been implicated in the nuclear retention of mRNAs with retained introns, which contain 5′ SS motifs, we examined whether TPR is required to retain reporter mRNAs with this element. We depleted TPR from U2OS cells using two lentiviral delivered shRNA (‘TPR A’ and ‘TPR D’; Figure 1A), and transiently transfected the *fushi tarazu* (*ftz*) reporter mini gene that either contains or lacks a ‘V5-His’ region (Figure 1B), which contains a 5′SS motif that inhibits nuclear export (26). In this case we used a version of the *ftz gene* that lacks an intron (*Δi*). We then visualized the reporter mRNA by fluorescence in situ hybridization (FISH) staining. Unexpectedly, we found that TPR-depletion did not inhibit the nuclear retention of the 5′SS motif-containing *ftz-Δi-V5-His* mRNA (Figure 1C, D, Supplementary Figure S1A). Instead, nuclear retention of this mRNA was enhanced (Figure 1C, D, Supplementary Figure S1A). We then moved the 5′SS motif closer to the 5′ end of the transcript, to more closely mimic where such motifs would be located in a transcript that fails to undergo splicing (Figure 1B). This reporter was only slightly retained in the nucleus (Figure 1C, D, Supplementary Figure S1A), possibly because the strength of the 5′SS is influenced by the surrounding sequence (26). Again, TPR-depletion only exacerbated nuclear retention (Figure 1C-D, Supplementary Figure S1A). Interestingly, the cytoplasmic accumulation of the *ftz* reporter that lacks a 5′SS motif (*ftz-Δi*) was also inhibited when TPR was depleted. Thus, TPR is required for the nuclear export of the intronless *ftz* reporter mRNA.

We next tested whether TPR-depletion affected the steady state RNA distribution of another reporter gene, *β-globin* (*βG*), with and without the V5-His region. Note that these constructs contain two introns (*βG-i*; Figure 1E). As was seen with *ftz*, TPR depletion did not inhibit, but rather enhanced, the nuclear retention of the V5-His-containing mRNA (*βG-i-V5-His*; Figure 1F-G, Supplementary Figure S1B). Surprisingly, in contrast to the *ftz* reporter, the distribution of *βG-i* was not significantly affected by TPR-depletion (Figure 1F, G, Supplementary Figure S1B).

**Figure 2.**
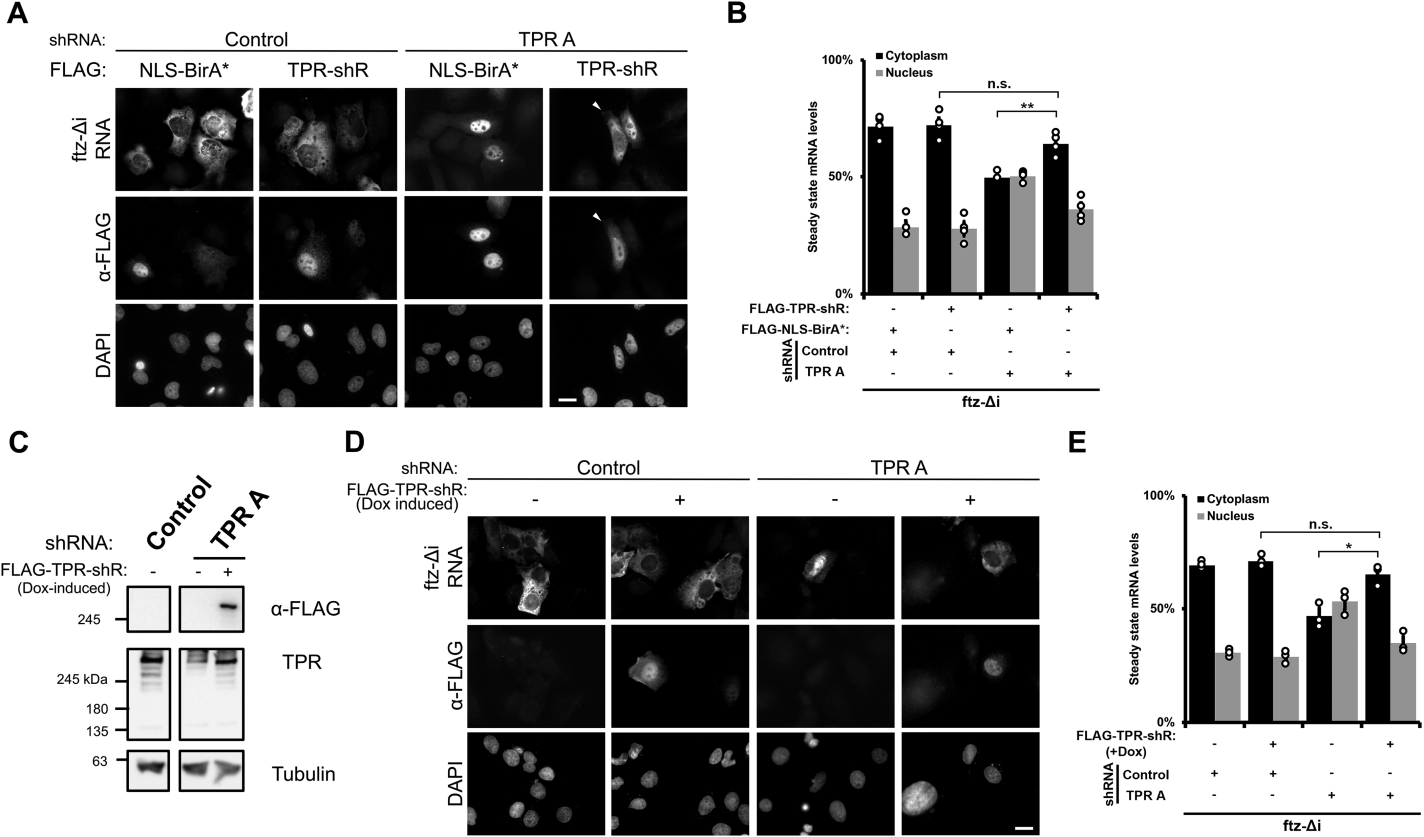
Re-expression of shRNA resistant FLAG-tagged TPR restores the cytoplasmic accumulation of TPR-sensitive reporter RNAs. (**A**, **B**) Cells were treated with either control or ‘TPR A’ shRNA and then co-transfected with *ftz-Δj* and either shRNA-resistant FLAG-tagged TPR (FLAG-TPR-shR) or FLAG-NLS-BirA*. Representative images show that re-expression of FLAG-TPR-shR restores the cytoplasmic accumulation of the *ftz* reporter RNA in TPR-depleted cells. RNA localization of the *ftz* reporter was only quantified in FLAG-positive cells, similar to Figure 1C with each bar representing the average and standard error for three independent experiments, each experiment consisting of at least 30 cells. Student t-test was performed in Figure 2B and D, *P < 0.05, **P < 0.01. (**C–E**) Flp-In U2OS cells that have an integrated shRNA-resistant FLAG-tagged *TPR* gene (*FLAG-TPR-shR*) were treated with control or ‘TPR A’ shRNA to deplete endogenous TPR and then treated with doxycycline for 24 h to express the integrated gene. (**C**) Immunoblots of the cells demonstrating the depletion of endogenous TPR and the doxycycline-induction of FLAG-TPR-shR expression. (**D**, **E**) Cells with and without doxycycline treatment were transfected with *ftz* reporter and the mRNA distribution was monitored by FISH. Quantification in (E) was carried out similar to (B).

We next tested if re-expression of TPR could rescue nuclear export of the *ftz-Δi* reporter. To achieve this, we expressed an shRNA resistant FLAG-tagged version of TPR (FLAG-TPR-shR) that was not repressed by the TPR A shRNA (Supplementary Figure S2A, B). Over-expression of this construct rescued the nuclear export of the *ftz-Δi* mRNA in cells treated with shRNA against endogenous TPR (Figure 2A, B). In contrast, expression of a control FLAG-tagged nuclear protein (FLAG-NLS-BirA*) had no effect on *ftz-Δi* mRNA export (Figure 2A, B). Next, we generated a Flp-In U2OS cell line where the FLAG-TPR-shR gene was inserted at an FRT-recombination site that typically allows for doxycycline-induced expression at near endogenous levels. Upon doxycyline treatment for 24 h, we observed that FLAG-TPR-shR is expressed in shRNA-treated cells at levels close to endogenous levels (Figure 2C) and can be observed at the nuclear rim (Supplementary Figure S2B) just like endogenous TPR (Supplementary Figure S2A). Similar to the overexpression experiment, we found that the induction of FLAG-TPR-shR expression rescued the export of the *ftz-Δi* reporter mRNA (Figure 2C, D), further demonstrating that the effects of shRNAs against TPR did not have off-target effects that impact mRNA export.

From these results we conclude that although TPR is not required for the nuclear retention of mRNAs that have 5′SS motifs, it is required for the cytoplasmic accumulation of certain mRNAs.

### TPR depletion influences mRNA stability, and the rate of nuclear export

The cytoplasmic accumulation of mRNAs can be due to a variety of factors. To determine whether this was due to changes in mRNA decay and/or mRNA nuclear export, we monitored the fate of newly synthesized mRNAs. To generate a pulse of transcribed mRNA, we microinjected plasmids that contained the *ftz-Δi* reporter into control and TPR-depleted cells, which allows for the rapid production of a large number of mRNA molecules in a short period of time (62,63). After allowing expression for 20 min, we added α-amanitin to halt further transcription and then fixed cells at various time points and determined the amount of mRNA in the nucleus and cytoplasm by FISH.

When we compared the amount of FISH signal in the TPR-depleted versus the control-depleted cells we observed that ∼40% of the *ftz* mRNA signal was eliminated within the first hour after transcriptional shut-off, but afterwards the ratio remained relatively constant (Figure 1H). This observation is similar to what we observed with reporter mRNAs that contain nuclear retention elements (26). When the nuclear/cytosolic distribution of the mRNA at theses same time points were analyzed, we observed that the rate of mRNA nuclear export was greater in the control versus the TPR-depleted cells (Figure 1I). Note that past the 1 hr mark, most of the remaining *ftz* mRNAs had effectively evaded RNA decay, but were still not effectively exported and thus trapped in the nucleus.

To validate these findings, we next microinjected *in vitro* synthesized *ftz* mRNA. Note that this mRNA was capped and polyadenylated and contained a signal sequence coding region, which are all required for efficient export of microinjected mRNA in mammalian cells (24,30). We observed that this mRNA was well exported in control cells but nuclear retained in TPR-depleted cells (Figure 1J, Supplementary Figure S3).

From these experiments we conclude that TPR-depletion causes the destabilization and the nuclear retention of certain mRNAs.

### TPR is required for the efficient nuclear export of poly(A)-mRNA

Our results suggested that TPR-depletion should promote a general nuclear mRNA-retention phenotype. Indeed, two previous reports showed that TPR depletion lead to nuclear accumulation of poly(A) signal, suggesting that TPR is required for the nuclear export of a significant number of mRNAs (11,38). In agreement with this, we also found that depletion of TPR increased the nuclear accumulation of poly(A) staining (Figure 1K, L, Supplementary Figure S1C). Taken together, our results indicate that TPR likely regulates the nuclear export of many mRNAs.

### TPR regulates the nuclear export of a subset of endogenous mRNAs and lncRNAs but is not involved in the nuclear retention of unspliced mRNAs

**Figure 3.**
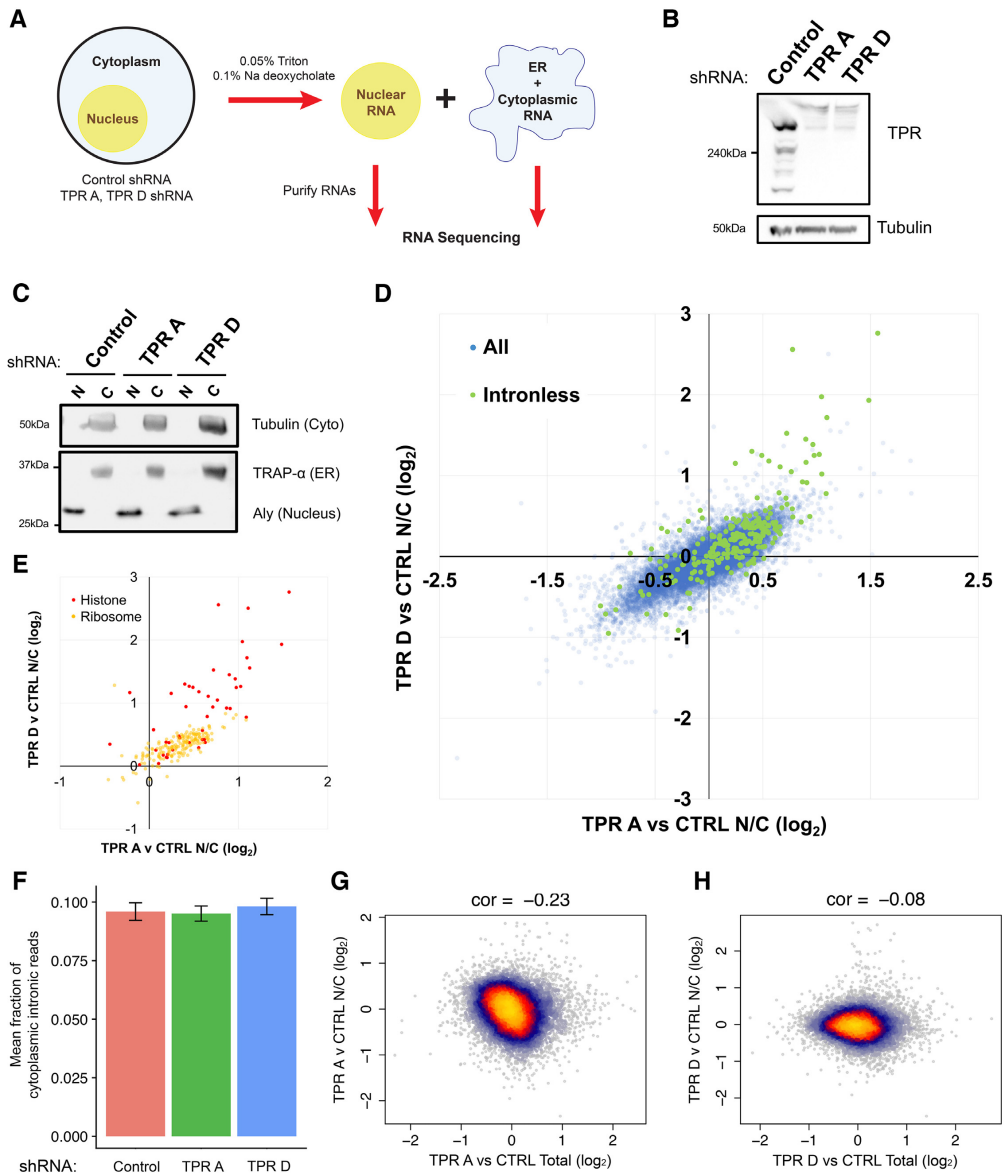
TPR-depletion inhibits the nuclear export of a subset of endogenous mRNAs. (**A**) Workflow for RNA Frac-Seq. Control- or TPR-depleted U2OS cells were fractionated into ‘nuclear’ and ‘ER/cytoplasmic’ fractions (see Material and Methods for more details). RNA from each fraction was purified and sent for RNA sequencing. (**B**) Immunoblot showing TPR-depletion for at least 96 h post-infection using two different lentiviral delivered shRNAs, ‘TPR A’ or ‘TPR D’. Tubulin is used as a loading control. (**C**) Immunoblot of nuclear ‘N’ or cytoplasmic ‘C’ fractions, probed with specific nuclear (Aly), ER (Trap-α) and cytoplasmic (Tubulin) protein markers. (**D, E**) Plot of the Log_2_ fold change of the nuclear/cytoplasmic ratio (TPR v CTRL N/C fold change) between TPR- and control-depleted cells for shRNA ‘TPR A’ (x-axis) or shRNA ‘TPR D’ (y-axis) for each mRNA. Positive values indicate an increase in the nuclear/cytoplasmic ratio in the TPR-depleted cells. All mRNA transcripts are shown in (**D**) with those transcribed from intronless genes (in green) being more nuclear enriched following TPR-depletion compared to those transcribed from other genes (in blue). Particular classes of mRNAs that have high nuclear enrichment upon TPR-depletion are shown in (**E**), including *histone* (in red) and *ribosomal* (in yellow) mRNAs. (**F**) Fraction of reads that map back to intronic regions of the genome are shown for cytoplasmic fractions from control and TPR-depleted cells. (**G, H**) Plot of the TPR-dependent changes in mRNA level (x-axis) versus TPR-dependent changes in nuclear export (TPR v CTRL N/C fold change) for shRNA ‘TPR A’ (**G**) or shRNA ‘TPR D’ (**H**) for each mRNA.

Next, we determined which RNAs require TPR for their nuclear export on a global scale using RNA Fractionation Sequencing (RNA Frac-Seq; Figure 3A). Briefly, we isolated nuclear and cytoplasmic/ER fractions from control- or TPR-depleted (shRNA 'TPR A’ or ‘TPR D’; Figure 3B, Supplementary Figure S4A) cells, then isolated and sequenced poly(A)+ selected RNAs. Each fraction was relatively free of cross contamination, as nuclear markers were well separated from cytosolic and ER-markers (Figure 3C). To control for changes in the transcriptome observed following TPR depletion, we isolated a ‘total RNA’ fraction and used this dataset to normalize against changes observed for both nuclear and cytoplasmic fractions.

To determine which RNAs require TPR for their nuclear export, we calculated the fold change of the RNA isolated from the nuclear and cytosolic compartments. A direct comparison of the nuclear to cytosolic fold change for the TPR-depleted cells against control shows that a subset of RNAs became nuclear enriched upon TPR-depletion (positive fold change value) and this change largely correlated between the two shRNA-treatments (Figure 3D). Upon a cursory inspection, we observed that many mRNAs transcribed from naturally intronless genes became nuclear retained upon TPR-depletion (Figure 3D, green data points). In particular, *histone* mRNAs, which are mostly intronless, were the most affected by TPR-depletion (Figure 3E, red data points). Although *histone* mRNAs undergo unique processing events, these mRNAs require TREX for their export (29,70). Another class of mRNAs that were affected by TPR-depletion were those that encode ribosomal proteins (Figure 3E, yellow data points). Unlike *histone* mRNAs, *ribosomal* mRNAs are generated from genes that have introns, suggesting that it was not only intronless mRNAs that required TPR for their efficient nuclear export.

Next, we analysed the effect of TPR-depletion on the export of mRNAs with retained introns. We observed no significant change in the fraction of cytoplasmic reads that mapped back to intronic regions (Figure 3F), suggesting that TPR is not required for the overall nuclear retention of unspliced mRNAs. It however remains possible that small subset of unspliced mRNAs are affected by TPR-depletion.

Recently it was observed that an acute TPR-depletion for 2 h led to a decrease in the level of certain mRNAs (35). Since we observed that nuclear retained mRNAs were substrates for decay (Figure 1I), we asked whether there was an inverse correlation between mRNA levels and nuclear retention in TPR-depleted cells. However, in our Fraq-Seq data we only saw a weak relationship between changes in mRNA levels and nucleoplasmic/cytoplasmic distribution (Figure 3G, H). Thus, if nuclear retention of an mRNA leads to its decay, this effect is only moderate at most. In addition, compensatory processes may also affect overall mRNA levels in our experiment. For example, we observed that TPR-depleted cells have higher overall levels of *histone* mRNAs (Supplementary Figure S4B), and this may explain why overall histone protein levels remain unchanged in TPR-depleted cells (Supplementary Figure S4C).

In summary, our Frac-Seq analysis of TPR-depleted cells indicate that the primary role of TPR is in regulating the nuclear export of a subset of mRNAs. Although it may regulate the nuclear retention of certain mRNAs, TPR-depletion did not promote a detectable increase in unspliced mRNAs in the cytoplasm.

### Short RNA transcripts require TPR for efficient nuclear export of their processed RNA products

**Figure 4.**
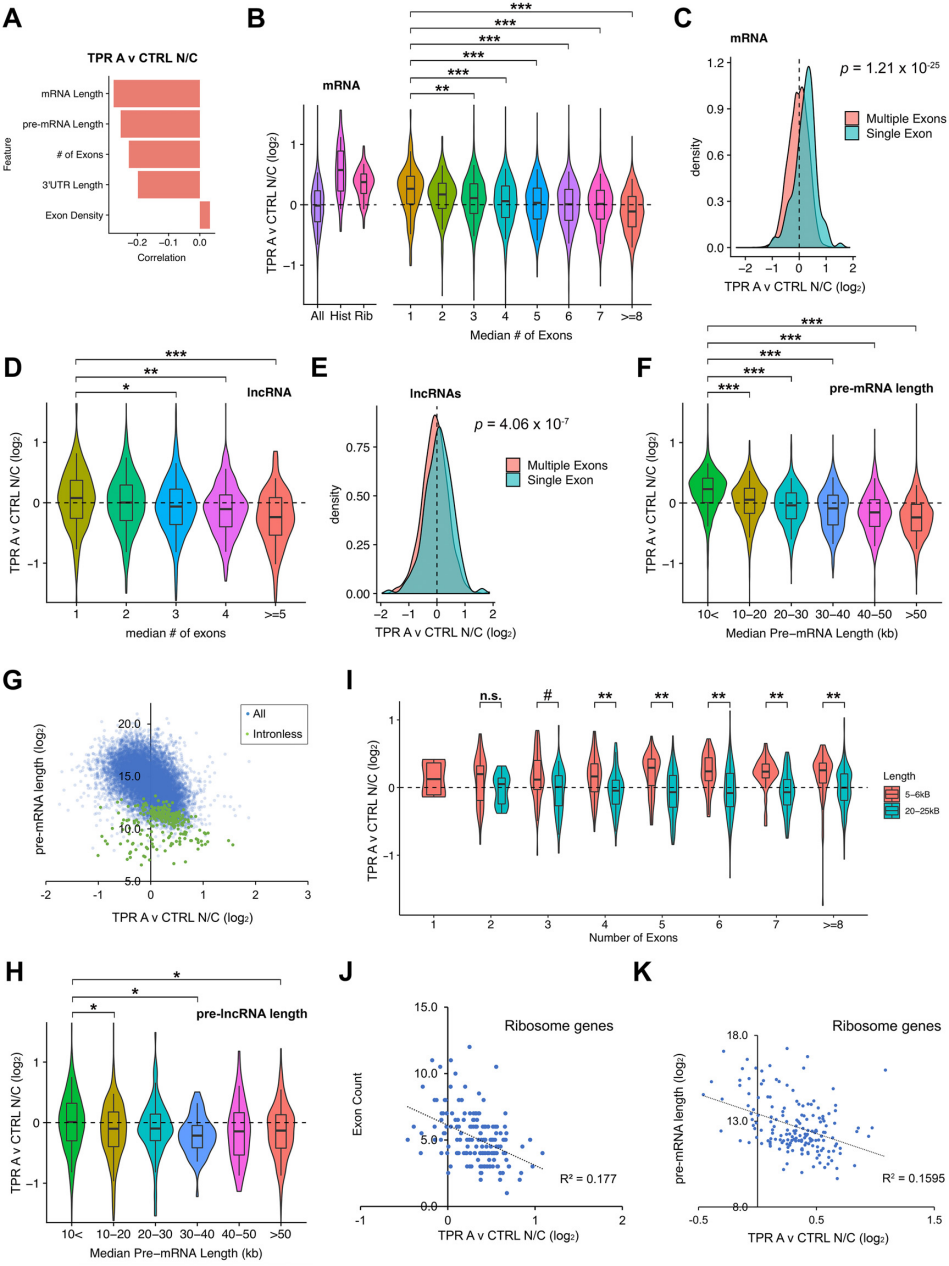
TPR-depletion inhibits the nuclear export of mRNAs and lncRNAs transcribed from short pre-mRNAs, including intronless and intron-poor genes. (**A**) Regression analysis of TPR-dependent nuclear export (TPR A v CTRL N/C fold change) and various RNA features. Note the inverse correlation between nuclear retention upon TPR-depletion (TPR A v CTRL N/C fold change) and gene length, pre-mRNA length, number of exons and 3′UTR length. (**B**) Violin plots of TPR-dependent nuclear export (TPR A v CTRL N/C fold change) for various classes of mRNAs. Note that mRNAs from *histone* and *ribosomal* genes are more nuclear enriched upon TPR depletion (positive fold change value) compared to all mRNAs. Also note that the nuclear export of mRNAs generated from few exons are more sensitive to TPR-depletion than other mRNAs. (**C**) Density plot showing that mRNAs from intronless genes are more nuclear enriched in TPR-depleted cells compared to those with multiple exons. (**D**) Violin plots of TPR-dependent nuclear export (TPR A v CTRL N/C fold change) for lncRNAs with various numbers of introns. Note that the nuclear export of lncRNAs generated from few exons are more sensitive to TPR-depletion than other lncRNAs. (**E**) Density plot showing that lncRNAs from intronless genes are slightly more nuclear enriched in TPR-depleted cells compared to those with multiple exons. (**F**) Violin plots of TPR-dependent nuclear export (TPR A v CTRL N/C fold change) for pre-mRNAs of varying lengths in 10 kb windows. Again, note the inverse correlation between pre-mRNA length and nuclear retention upon TPR-depletion. (**G**) Scatter plot of TPR-dependent nuclear export (TPR A v CTRL N/C fold change) and pre-mRNA length. Note that the inverse correlation between nuclear retention upon TPR-depletion and pre-mRNA lengths. Intronless mRNAs (green dots) are shorter in length compared to all other pre-mRNAs (blue dots) and are most dependent on TPR-dependent nuclear export. (**H**) Violin plots of TPR-dependent nuclear export (TPR A v CTRL N/C fold change) for pre-lncRNAs of varying lengths in 10 kb windows. The trend for pre-lncRNAs is similar to pre-mRNAs, but again not as strong. For details, see text. (**I**) Violin plots of TPR-dependent nuclear export (TPR A v CTRL N/C fold change) for pre-mRNAs that are 5–6 kb or 20–25 kb in length with various numbers of exons. (**J, K**) For mRNAs from ribosomal protein genes, the degree of TPR-dependent nuclear export (TPR A v CTRL N/C fold change) was plotted against the number of exons (**J**) or pre-mRNA length (**K**). Note the weak correlation in each case. Mann-Whitney-Wilcoxon statistical tests with Bejamini–Hochberg adjustment for multiple comparisons was performed for B, D, F, H and I, ^#^*P* > 0.05, * *P* < 10^−3^, **P < 10^−5^ and ***P < 10^−10^.

Next, we performed a linear regression analysis to fully explore the features shared by mRNAs that require TPR for their nuclear export (Figure 4A, Supplementary Figure S5A). Validating our initial observations, we found that the number of exons negatively correlated with TPR-dependent nuclear export (Figure 4A, B, Supplementary Figure S5A, B). In addition, we observed that mRNA and pre-mRNA length also negatively correlated with TPR-dependent nuclear export. This is not surprising as *ribosomal* and *histone* mRNAs which are both TPR-sensitive (Figure 4B, Supplementary Figure S5B) tend to be short as their encoded proteins are all smaller than 35kDa, whereas the average human protein is ∼50 kDa. Furthermore, mRNAs generated from intronless genes tend to be shorter than other mRNAs. Moreover, since the primary contributor to gene length is introns, it is also to be expected that intronless and intron-poor genes, tend to be shorter than other genes.

In agreement with the idea that splicing (or the lack of splicing) plays a role in TPR-dependent mRNA nuclear export, we found that mRNAs that contained fewer introns had a higher nuclear/cytoplasmic ratio upon TPR-depletion (Figure 4B, C, Supplementary Figure S5B, C). This was mostly due to a drop in the cytoplasmic level of these transcripts (Supplementary Figures S6A–D and S7A–D). We also saw an increase in the nuclear/cytoplasmic ratio for lncRNAs that had fewer introns (Figure 4D, S5D), however in general there was less of an overall difference between single and multiple exon lncRNAs in aggregate (Figure 4E, Supplementary Figure S5E), perhaps because lncRNAs in general have few introns. Interestingly, when the level of lncRNAs in the nucleus and cytoplasm were assessed independently, there was a drop in both compartments (Supplementary Figures S6E–H and S7E–H). This suggested that TPR-depletion caused an overall drop in lncRNA stability.

We next determined whether pre-mRNA length plays a role in TPR dependent nuclear export. We observed that the shorter a pre-mRNAs was, the more it was dependent on TPR for its nuclear export (Figure 4F–G, Supplementary Figure S5F–G). In agreement with this, mRNAs produced from short transcripts were more nuclear (Supplementary Figure S8A–B) and less cytoplasmic (Supplementary Figure S8C-D) in TPR-depleted cells compared to those produced from long transcripts. A similar relationship was seen with lncRNAs (Figure 4H, Supplementary Figures S5H and S8E–H).

Since there is a correlation between pre-mRNA length and number of exons, we next wanted to disentangle these two features. To limit the amount of variation in length, we analyzed short (5–6 kb) and long (20–25 kb) pre-mRNAs (note that the median length of the pre-mRNAs in our dataset is ∼21 kb). We found that the export of mRNA produced from shorter pre-mRNAs was more sensitive to TPR-depletion than longer transcripts, and this was true regardless of the number of exons (Figure 4I, Supplementary Figure S5I). When pre-mRNA length was controlled for, there was little correlation between exon number and TPR dependence for nuclear export (Figure 4I, Supplementary Figure S5I). These results suggest that pre-mRNA length plays a major role in determining whether a given mRNA is TPR-dependent independent of splicing. The effect of pre-mRNA length explains why mRNAs encoding ribosomal proteins are affected as they tend to have short introns (71). In line with this, we saw a slight negative correlation between either number of exons or pre-mRNA length with TPR-dependence for nuclear export for ribosomal protein mRNAs (Figure 4J–K, Supplementary Figure S5J–K).

Overall, our data suggest that pre-RNA length plays a major role in determining whether the efficient nuclear export of a given RNA requires TPR. Importantly, TPR was not only required for the efficient export of mRNAs transcribed from intronless and intron-poor genes, which tend to have shorter pre-mRNAs, but also from mRNAs produced from short transcripts, regardless of how many splicing events these mRNAs experience. This agrees with our reporter data, where the nuclear export of the single exon *ftz* mRNA is TPR-sensitive, while the export of the three exon *βG-i* mRNA, produced from a longer pre-mRNA, is TPR-insensitive.

### TPR is required for the efficient export of the *NORAD* lncRNA and *Histone1* mRNA

**Figure 5.**
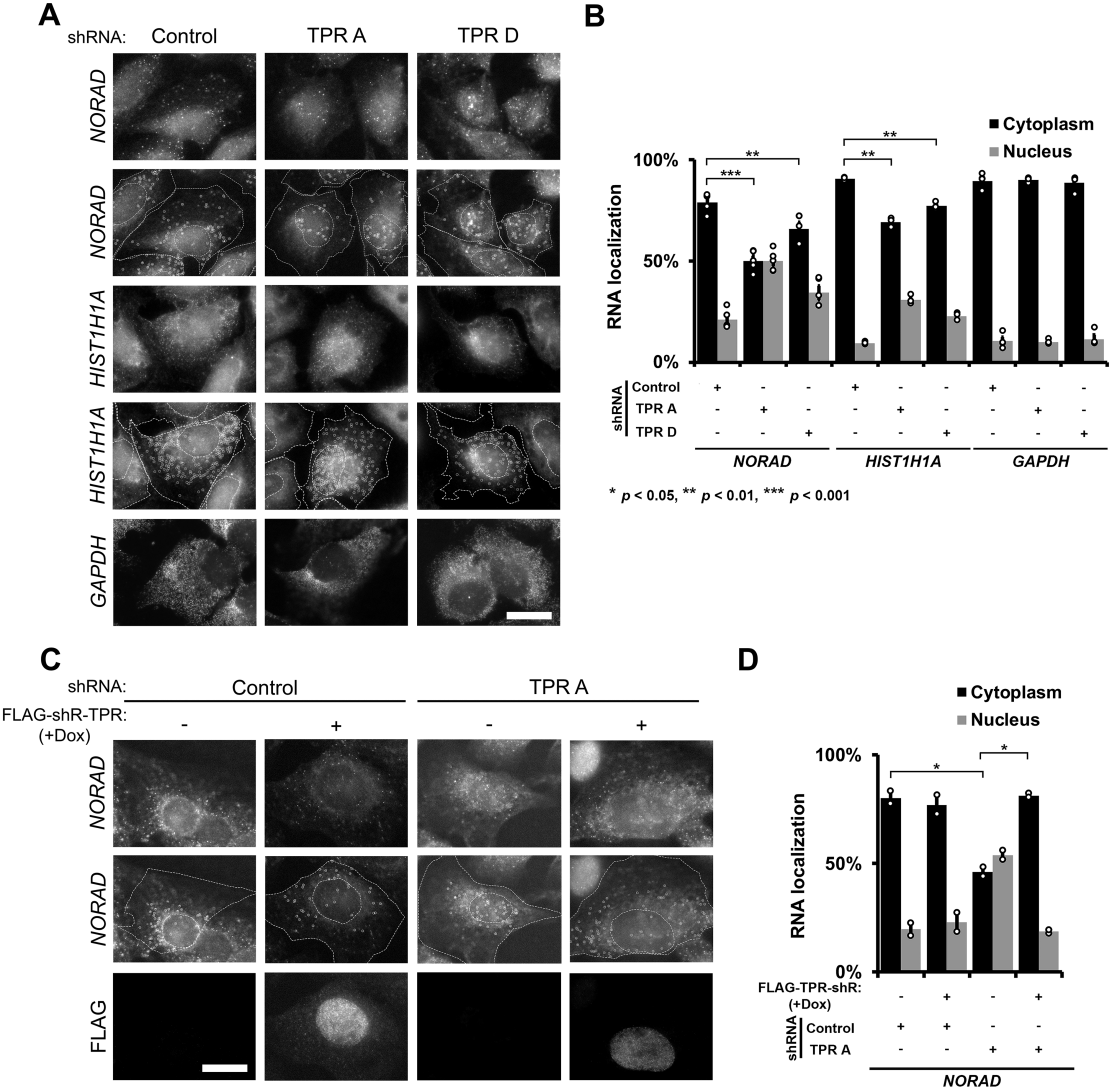
TPR is required for the efficient export of *NORAD* lncRNA or *HIST1H1A* mRNA. (A, B) U2OS cells were treated with shRNAs against TPR or scrambled control, then fixed and stained for *NORAD* lncRNA, *HIST1H1A* mRNA or *GAPDH* mRNA by single molecule FISH. Examples are shown in (**A**) with the top two rows showing the exact same images except that in the second row the cell and nuclear boundaries are delimited by dashed lines and single *NORAD* lncRNAs or *HIST1H1A* mRNAs are circled. The nuclear cytoplasmic distribution of single mRNAs in various conditions is quantified in (**B**) with each bar representing the average and standard error of at least three independent experiments, each experiment consisting of at least 50 cells. Student's *t*-test was performed, * *P* < 0.05, ** *P* < 0.01, *** *P* < 0.001. (**C, D**) Re-expression of FLAG-TPR-shR restores cytoplasmic accumulation of *NORAD* lncRNA in TPR depleted cells. FLAG-TPR-shR expression was induced by doxycycline treatment in a Flp-In U2OS cell line. Quantification in (D) is as for (B).

Our RNA Frac-Seq results suggest that TPR is required for the efficient nuclear export of mRNAs and lncRNAs that are produced from pre-mRNAs that are either short and/or undergo fewer splicing events. To independently assess this, we used single molecule FISH to monitor the distribution of the two RNAs: *NORAD* lncRNA, which is primarily found in the cytoplasm of U2OS cells, and *Histone 1* (*HIST1H1A*) mRNA. *NORAD* is 5378 nt long and has only a single exon, and thus would be expected to be dependent on TPR for its nuclear export. *HIST1H1A* mRNA is also intronless and is 780 nt long. Indeed, we found that upon TPR-depletion, the nuclear level of both *NORAD* and *HIST1H1A* rose 2–3-fold (Figure 5A–B). In contrast, the distribution of *GAPDH* mRNA, which is 1292 nt long and contains 9 exons, was mostly cytoplasmic in both control and TPR-depleted cells (Figure 5A–B). Interestingly the *GAPDH* pre-mRNA is generated from a 3885 nt long pre-mRNA, which is much shorter than the *NORAD* mRNA. Thus, although pre-mRNA length may contribute, other factors, such as splicing and the presence of *cis*-elements likely play a role.

We then repeated these experiments in the U2OS cell line containing the doxycycline-inducible *FLAG-TPR-shR* gene. Again, depletion of endogenous TPR caused the nuclear accumulation of *NORAD* lncRNA, while induction of FLAG-TPR-shR expression restored nuclear export of *NORAD* lncRNA (Figure 5C–D), thus validating that this effect is due to depletion of TPR.

In summary, these data support the idea that mRNAs and lncRNAs produced from intronless or intron-poor genes require TPR for their efficient export.

### Spliced reporter mRNAs, and reporter mRNAs synthesized from longer pre-mRNAs, are less dependent on TPR for their nuclear export

**Figure 6.**
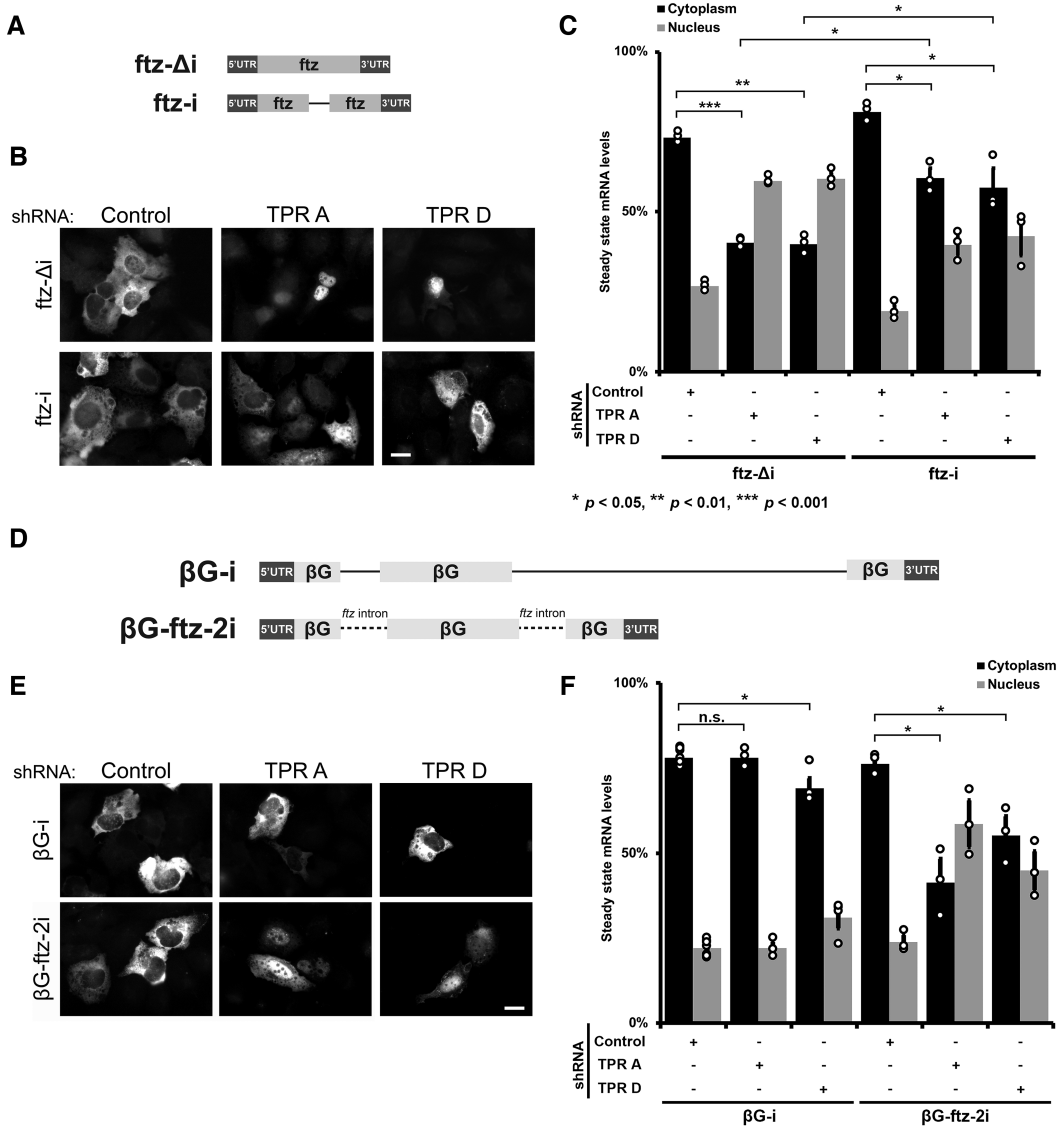
Spliced reporter mRNAs are less dependent on TPR for their nuclear export. (**A**) Schematic of the *ftz* reporter with and without an intron (±*i*). (**B, C**) Control- or TPR-depleted cells were transfected with various *ftz* reporter plasmids. 18–24 h later the cells were fixed and the mRNA was visualized by FISH. Representative images are shown in (**B**), quantification is shown in (**C**). Scale bar = 10 μm. Each bar is the average and standard error of three independent experiments, each experiment consisting of at least 60 cells. Note that TPR-depletion caused nuclear accumulation of the *ftz-Δi* mRNAs, but not the spliced *ftz-i* mRNAs. (**D**) Schematic of *βG* reporters with the *ftz* intron inserted in place of the endogenous *βG* introns. (**E, F**) Control- or TPR-depleted cells were transfected with various *βG* reporter plasmids. 18–24 h later the cells were fixed and the mRNA was visualized by FISH. Representative images are shown in (**E**), and the nuclear and cytoplasmic ratios were quantified in (**F**). Scale bar = 10 μm. Each bar is the average and standard error of three independent experiments, each experiment consisting of at least 60 cells. Note that TPR-depletion caused nuclear accumulation of *βG* mRNAs with the *ftz* introns, *βG-ftz-2i*, but not the *βG* reporter with endogenous introns. Student t-test was performed in C and F, * *P* < *P* < 0.05, ** *P* < 0.01, *** *P* < *P* < 0.001.

Our Fraq-Seq analysis suggests that there are at least two main factors that determine whether an mRNA requires TPR for its efficient export: the degree of splicing and the pre-mRNA length. To further confirm that splicing determines whether an mRNA requires TPR for its efficient nuclear export, we measured the nuclear/cytoplasmic distribution of the *ftz* reporter mRNA synthesized from genes that lacked (*ftz-Δi*) or contained (*ftz-i*) an intron (Figure 6A). Note that this intron is quite small (141 nt) and contributes minimally to the transcript length. Indeed, we found that a mRNA produced from *ftz-i* was less nuclear retained in TPR-depleted cells than *ftz-Δi*, suggesting that in this case the presence of one intron partially relieved the need for TPR for efficient export (Figure 6B–C). Interestingly, this mRNA was partially nuclear retained in TPR-depleted cells when compared to control cells, indicating that the need for TPR was not totally bypassed.

Next, we tested whether the length of the pre-mRNA contributed to whether nuclear export was TPR-sensitive or not. To accomplish this we replaced the two introns of the *βG-i* reporter mRNA, whose export does not require TPR, with the introns of our *ftz* reporter RNA (*βG-ftz-2i*, Figure 6D). The pre-mRNA length generated from this *βG-ftz-2i* is 738 nucleotides long, compared to *βG-i*, which is 1424 nucleotides long. When we expressed the *βG-ftz-2i* reporter in TPR depleted cells, the mRNA reporter was more nuclear retained than *βG-i* mRNA (Figure 6E–F).

Our results suggest that both splicing and intron length (and by extension pre-mRNA length), are major determinants of whether an mRNA requires TPR for its efficient nuclear export. These data corroborate findings from our Frac-Seq experiments.

### mRNAs that are retained in TPR-depleted cells, accumulate in nuclear speckles

**Figure 7.**
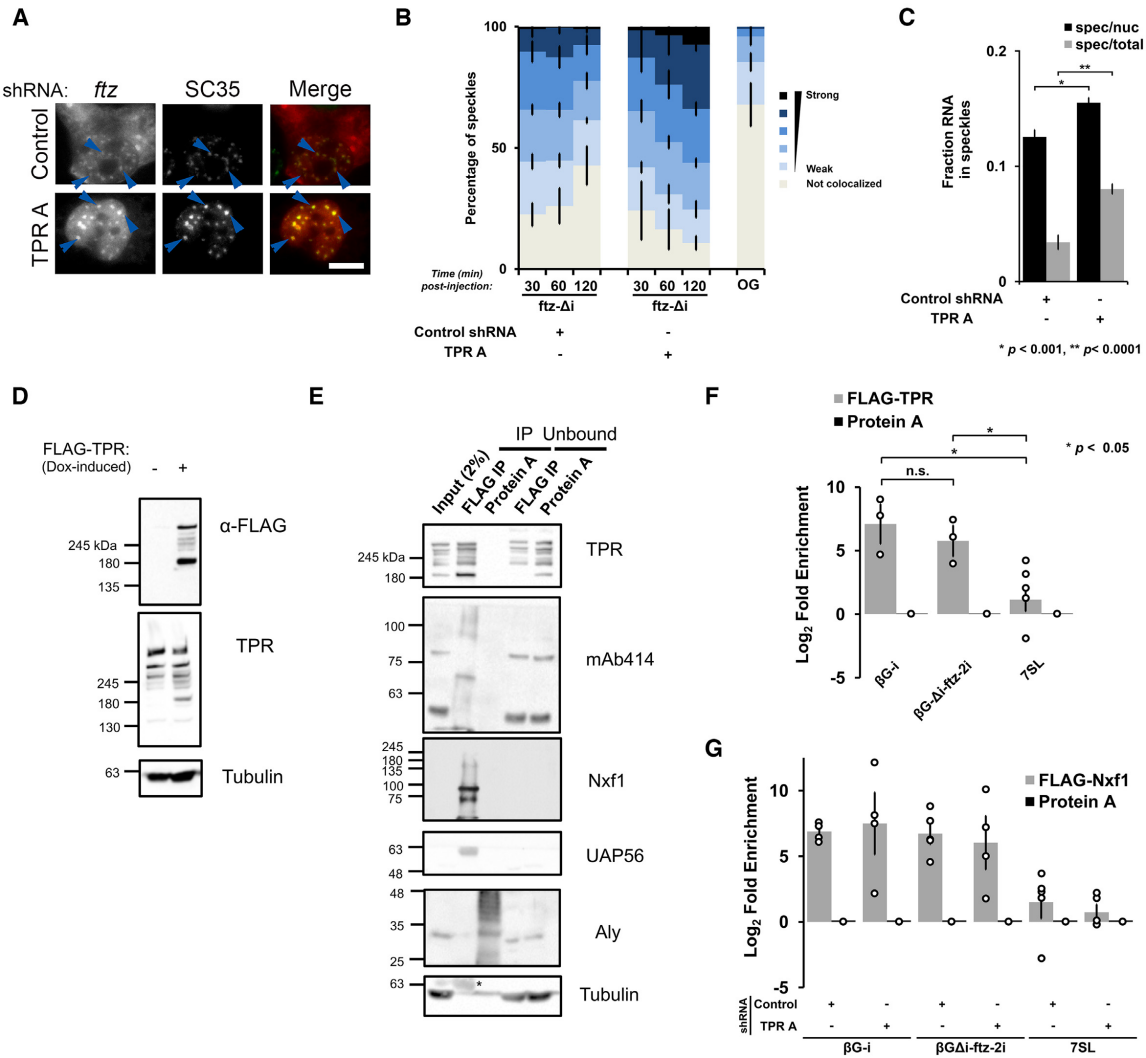
mRNAs that are retained in TPR-depleted cells accumulate in nuclear speckles and are bound to Nxf1. (**A–C**) Control- or TPR-depleted cells were microinjected with plasmid *ftz-Δi* reporter plasmids. After the indicated times, cells were fixed and stained for *ftz* mRNA by FISH and for the speckle marker SC35 by immunofluorescence. (**A**) An example of cells fixed 2 h post-injection with each row represents a single field of view with blue arrow pointing to examples of *ftz*/SC35 co-localization. Scale bar = 10 μM. (**B**) The degree of *ftz*/SC35 co-localization by Pearson correlation coefficient analysis was quantified as described in (24). As a control, the co-localization of microinjected 70 kD dextran conjugated to Oregon Green (‘OG’) with SC35 speckles was also tabulated. Each bar is the average and standard error of three independent experiments, each consisting of 150–200 nuclear speckles (see Materials and Methods for more details). (**C**) The amount of *ftz-Δi* mRNA present in nuclear speckles (defined by the brightest 10% pixels in the nucleus, using SC35 immunofluorescence - described in (24)) as a percentage of either the total nuclear (‘spec/nuc’) or total cellular (‘spec/total’) mRNA level in cells 1 h post-microinjection. Each data point represents the average and standard error of the mean of 10–20 cells. (**D**) Western blot showing that FLAG-TPR was successfully expressed following doxycycline treatment for 4 h. (**E**) Flp-In FLAG-TPR U2OS cells were treated with doxycycline for 4 h, then lysed and immunoprecipitated with anti-FLAG M2 or protein A beads. 2% of the input lysate, Immunoprecipitates (IP), or 10% of the unbound fraction were separated by SDS-PAGE and immunoblotted using the indicated antibodies. Note the presence of IgG heavy band (*) in the anti-tubulin immunoblot. (**F**) Same as (E), except following doxycycline treatment, the Flp-In FLAG-TPR U2OS cells were transfected with either *βG-i* or *βG-ftz-2i* reporter plasmids for 18 to 24 h. These cells were then lysed and immunoprecipitated as in (E). The amount of *βG* mRNA and the *7SL* ncRNA in the immunoprecipitates were quantified by RT-qPCR. Each bar is the average and standard error of at least three independent experiments. Student t-test was performed, * *P* < 0.05. (**G**) Control- or TPR-depleted cells were co-transfected with FLAG-Nxf1 and either the *βG-i* or *βG-ftz-2i* reporter plasmids for 18–24 h, then lysed and immunoprecipitated with anti-FLAG M2 beads or protein A beads. The amount of *βG* and the *7SL* ncRNA in the immunoprecipitates were quantified by RT-qPCR. Each bar is the average and standard error of at least four independent experiments.

Previously, we and others have observed that in cells depleted of TREX components, mRNAs become retained in nuclear speckles (24,28,72), which are small phase-separated regions of the nucleoplasm that are enriched in splicing factors and TREX components (73). Nuclear speckles have been shown to function as sites of post-transcriptional splicing (74,75). Furthermore, work from our lab and others suggests that many mRNPs are targeted to nuclear speckles, where they either undergo a maturation process to become competent for nuclear export, or are nuclear retained (24,26,28,^56^,^72^,^76^). Indeed, it was previously seen that TPR-depletion led to the enrichment of total poly(A) mRNA with nuclear speckles (35,38). To monitor how newly synthesized mRNAs trafficked through speckles, we microinjected plasmid containing the *ftz-Δi* reporter gene, whose mRNA transits through nuclear speckles (24), and monitored the distribution of this mRNA by FISH and nuclear speckles using antibodies to SC35. In control cells, we observed that the newly synthesized *ftz* mRNA quickly entered into speckles, but over time it left these structures as it began to be exported. In TPR-depleted cells, *ftz* mRNA continued to accumulate into speckles over the duration of the time course (Figure 7A–C). These observations suggest that TPR may be involved in the release of these mRNAs from nuclear speckles prior to mRNA export. This could be due to direct (i.e. TPR interacting with mRNPs and affecting their maturation) or indirect (e.g. TPR affecting the nuclear/cytoplasmic trafficking of proteins that make up the mRNP or speckles) effects.

### TPR interacts with mRNAs

We next investigated whether TPR associates preferentially with mRNAs whose nuclear export is dependent on this protein. We transfected our reporter constructs in a U2OS cell line expressing FLAG-TPR from an FRT-integrated gene and performed a FLAG immunoprecipitation from the cell lysates. Note that this construct is expressed at levels slightly below that of endogenous protein (Figure 7D) and is present in both the nuclear rim and nucleoplasm (Supplementary Figure S2C). FLAG immunoprecipitates contained various nuclear pore proteins (as detected by mAb414, which recognizes FG-containing Nups), the nuclear transport receptor Nxf1, and the TREX-component member UAP56 (Figure 7E). The TREX component Aly and tubulin were not significantly enriched in the immunoprecipitate over the control precipitate (Protein A). When cells expressed the two versions of the *βG* mRNA, one whose nuclear export is insensitive (*βG-i*) and a second whose export is sensitive (*βG-ftz-2i*) to TPR-depletion, both mRNAs were enriched in the immunoprecipitate (Figure 7F). In contrast, the 7SL non-coding RNA, which utilizes exportin-5 for its nuclear export (77), was not significantly enriched (Figure 7F). Thus, it appears that TPR associates with both mRNAs that require it for export (*βG-i*) and those that do not (*βG-ftz-2i*). This promiscuous interaction profile may simply reflect the fact that all of these mRNAs must pass through the pore. As TPR also binds to many RNA-binding proteins, it is not clear whether TPR associates directly or indirectly to mRNAs.

Following up on the observation that TPR binds to Nxf1 and other mRNA export factors, we tested whether Nxf1 binding to mRNA is dependent on TPR. We expressed FLAG-tagged Nxf1 and performed RNA immunoprecipitation experiments in TPR-depleted and control cells. We found that TPR-depletion did not affect Nxf1-binding to either *βG-i* or *βG-Δi-ftz-2i* mRNA (Figure 7G). These data suggest that TPR does not help to recruit Nxf1 to the mRNA, but instead acts downstream from this step.

## DISCUSSION

Here, we show that the nuclear basket protein TPR is required for the nuclear export of mRNAs and lncRNAs that are produced from intronless and intron-poor genes that tend to be short. In line with this, we find that the nuclear export of reporter mRNAs becomes more TPR-dependent when their pre-mRNA length shortens and when they are produced from pre-mRNAs that have fewer introns. Furthermore, these mRNAs are associated with Nxf1 even when TPR is depleted, suggesting that TPR acts at a very late step in the export pathway. Finally, we observe that upon TPR-depletion these mRNAs become enriched in nuclear speckles, indicating that TPR may influence how mRNAs may traffic through these structures. There has been considerable debate surrounding the role of splicing in nuclear mRNA export (25,26,56). What is clear is that intronless mRNAs are exported in a TREX-dependent manner (23,24,30,78), but that they are subject to nuclear retention if they contain certain elements (25). Our work demonstrates that these mRNAs also have additional requirements for their export. In contrast, spliced mRNAs, which are generated from longer pre-mRNA transcripts, appear to have slightly different requirements.

Recently, it was found that acute depletion of TPR led to a decrease in the total levels of certain mRNAs (35). This is consistent with the hypothesis that nuclear retained mRNAs are degraded, as is the case with our reporter mRNA (Figure 1H). However, we found that in TPR-depleted cells changes in global RNA levels did not correlate with changes in nuclear/cytoplasmic distribution (Figure 3G–H). Moreover, since the depletion in this previous study was only for 2 h, a much shorter time span than the average half-life of mRNAs in mammalian cells (5–10 h (79)), it is unlikely that this treatment would have much impacted the overall distribution of most mRNAs. It remains possible that the short-term depletion of TPR may enhance mRNA stability, however whether this is associated with nuclear retention needs to be tested. Interestingly, in our experiments the prolonged depletion of TPR led to an increase in the total levels of certain nuclear retained mRNAs, such as those encoding histones. This may be a compensatory mechanism to ensure that overall histone production remains constant. Another caveat is that prolonged depletion of TPR in our experiments may alter the levels or distribution of factors required for the export of mRNAs transcribed from short intron-poor genes.

Our data also indicate that TPR is not required for the nuclear retention of 5′SS motif-containing RNAs. Indeed, we found that if anything, mRNAs with these motifs were even more nuclear retained after TPR-depletion. Although it is possible that TPR may retain mRNAs that contain other elements found in introns (such as the 3′ splice site), we did not find evidence for the increased export of intron-containing transcripts in our Fraq-Seq experiments (Figure 3F). Previous studies that implicated TPR in the retention of intron-containing mRNAs, studied viral mRNAs which have additional nuclear retention elements besides the 5′SS motif (56). It thus remains unclear how TPR affects the nuclear retention of these RNAs. In yeast, it had been reported that the TPR-paralogues, Mlp1 and Mlp2, are required for efficient nuclear retention of mRNAs with unspliced introns (52). Importantly, these studies did not investigate mRNA localization directly, but instead measured the production of a reporter protein which could only be produced from an intron-containing mRNA and comparing this to the expression of a protein from a control construct that lacks an intron. It remains possible that the deletion of Mlp1 and/or Mlp2 affected the export of both the reporter and the control mRNA to different extents, thus confounding any interpretations. Alternatively, Mlp1/2 may have roles in mRNA retention in yeast that are not conserved in metazoans. The role of TPR in the nuclear retention of intron-containing mRNAs in human cells has also been investigated only indirectly through the expression of protein from intron-containing reporter mRNAs in comparison to some control (50,51). Again, since our data suggest that TPR-depletion has a major effect on the export of intronless mRNAs, which would affect the export of most of the control mRNAs used in these experiments, the results from these past studies may be uninterpretable. Reanalyzing these experiments by directly monitoring the nuclear and cytoplasmic levels of these mRNAs may resolve these apparent conflicts.

Interestingly, one TPR-interacting protein, TARBP2, was found to bind and inhibit the splicing of certain introns that contain TARBP2-binding structural elements (80). The authors proposed that TPR may help to retain these unspliced mRNAs in the nucleus where they are subsequently degraded. When we compared our TPR Frac-Seq dataset, with TARBP2-bound mRNAs (known as the ‘TARBP2 regulon’ (81)) these mRNAs are slightly more cytoplasmic upon TPR depletion (Supplementary Figure S9A, B), suggesting that TPR may indeed promote their nuclear retention; however, since these mRNAs on average have more exons (Figure S9C) and their mRNAs are longer in length (Figure S9D) than other mRNAs, it may also be equally likely that these mRNAs are overrepresented in the cytosol of TPR-depleted cells due to their features which are unrelated to intron-retention and TARBP2-association.

The mechanism by which TPR influences nuclear export needs to be elucidated. It remains unclear why TPR would affect the nuclear export of mRNAs produced from short pre-mRNAs, which includes intronless and intron-poor primary transcripts. Short and long pre-mRNA transcripts may be loaded with different RNA-binding proteins during transcription and these factors may render an mRNA more or less sensitive to TPR-depletion. Many nucleoporins have been shown to be also present in the nucleoplasm where they have been shown to bind to chromatin and affect transcription (47,49,82,83). Interestingly the ability of TPR to influence how mRNAs traffic through nuclear speckles and to interact with reporter mRNA and components of the TREX complex may suggest that TPR initiates contact with the mRNA before it reaches the nuclear pore. In flies, it has been shown that the TPR homolog, Megator, and a second component of the nuclear basket, Nup153, binds to chromatin, forming nucleoporin-associated regions that are dominated by markers of active transcription (48). Similarly, in human cells TPR binds to the promoter region of *HSP104*, and this interaction promotes the association of TPR with the *HSP104* mRNA and is required for its proper nuclear export (84). It is likely that these interactions do not happen in the vicinity of the pore, but within the interior of the nucleoplasm (48), where a substantial fraction of TPR resides (37,85).

Despite the fact that early steps in mRNA synthesis/processing determine whether a given transcript requires TPR for its export, TPR likely affects a late step of mRNA nuclear export. In TPR-depleted cells, affected mRNAs are still associated with Nxf1 and enriched in speckles. Interestingly, it has been observed that intronless mRNAs, which appear to transit through nuclear speckles to gain export competency (24,28), are present in the nucleoplasm but not enriched in nuclear speckles in Nxf1-depleted cells (28). Importantly these same mRNAs are found in speckles in cells depleted of UAP56 alone or in UAP56/Nxf1 co-depleted cells (28). Our new results would indicate that Nxf1 gets recruited to the mRNA in speckles in a TPR-independent manner. In sum, these observations suggest that TREX and TPR play a role in nuclear speckle egress while Nxf1, which is recruited to mRNAs either in speckles or prior to speckle entry, acts on mRNAs after speckle egress but prior to export. This would be consistent with data indicating that Nxf1 may also associate with mRNAs co-transcriptionally (86).

TPR may influence export by interfacing with components of TREX-2. In the absence of TPR, proteins in this complex no longer localizes to the nuclear pore (11,14,17,35). Interestingly, mRNAs that require GANP for their nuclear export tend to be shorter in length and contain few introns (14), mirroring our Frac-Seq analysis of TPR-depleted cells. Indeed, GANP-depletion led to drop in the export of histone mRNAs (35). However, there is some evidence to suggests that GANP may also function independently of TPR. First, nuclear accumulation of poly(A)+ RNAs was further enhanced in TPR/GANP double-depleted cells when compared to the single depleted cells (14) suggesting that these two factors act on different mRNAs. Second, we previously demonstrated that GANP-depletion inhibited the export of spliced *βG-i* (36), which we now show is exported in TPR-depleted cells (Figures 1F–G and 6E). It remains possible that GANP is required for both TPR-dependent and TPR-independent export. GANP itself has several FG-repeats that may facilitate its partitioning into the central region of the nuclear pore, which is thought to form a phase-separated domain where FG-Nups and nuclear transport receptors partition (87). In light of this, it is possible that TPR may facilitate the interaction of certain mRNPs with GANP, and thus enhance passage through the pore. In GANP-depleted cells, mRNAs that lose the ability to be exported accumulate in nuclear speckles (36), thus perhaps GANP-recruitment to the transcript is also essential for nuclear speckle egress.

While this manuscript was being revised, a report was published from the Ulitsky group describing that TPR-depletion affected the export of mostly intronless and intron-poor mRNAs and lncRNAs (88). Furthermore, in agreement with our observations, the Ulitsky group found that TPR-depletion did not affect the retention of unspliced mRNAs. Generally, the two studies’ results largely agree, although the Ulitsky group did not analyze whether the length of the pre-mRNA correlated with TPR-dependence on export. Data from the Ulitsky group indicates that both unspliced and spliced mRNAs require the TREX complex, although the later group of transcripts is more affected by TREX-depletion. Overall, it appears that TPR plays a significant role in mRNA export and less so for mRNA retention. Further work will need to be done to determine the exact role of TPR in these processes and how it interfaces with Nxf1 and TREX to promote RNA nuclear export.

## DATA AVAILABILITY

RNA Fraq-Seq data were deposited in GEO database as GSE135537. Data can be visualized using the following track hub: http://genome.ucsc.edu/cgi-bin/hgTracks?db=hg19&lastVirtModeType=default&lastVirtModeExtraState=&virtModeType=default&virtMode=0&nonVirtPosition=&position=chr12%3A6645614%2D6646097&hgsid=750366017_JoxA2qgpq9SK9Lp7qBWY5LM5Tuu4.

## Supplementary Material

gkaa919_Supplemental_File

## Notes

Present address: Eric J. Wolf, New England Biolabs, Ipswich, MA, USA.
