# Supplementary material for: TPR is required for the efficient nuclear export of mRNAs and lncRNAs from short and intron-poor genes
Source: Nucleic Acids Res. 2020 Oct 22;48(20):11645–63. doi: 10.1093/nar/gkaa919 (PMC7672458; doi:10.1093/nar/gkaa919)
Supplement: gkaa919_Supplemental_File [file gkaa919_supplemental_file.pdf]

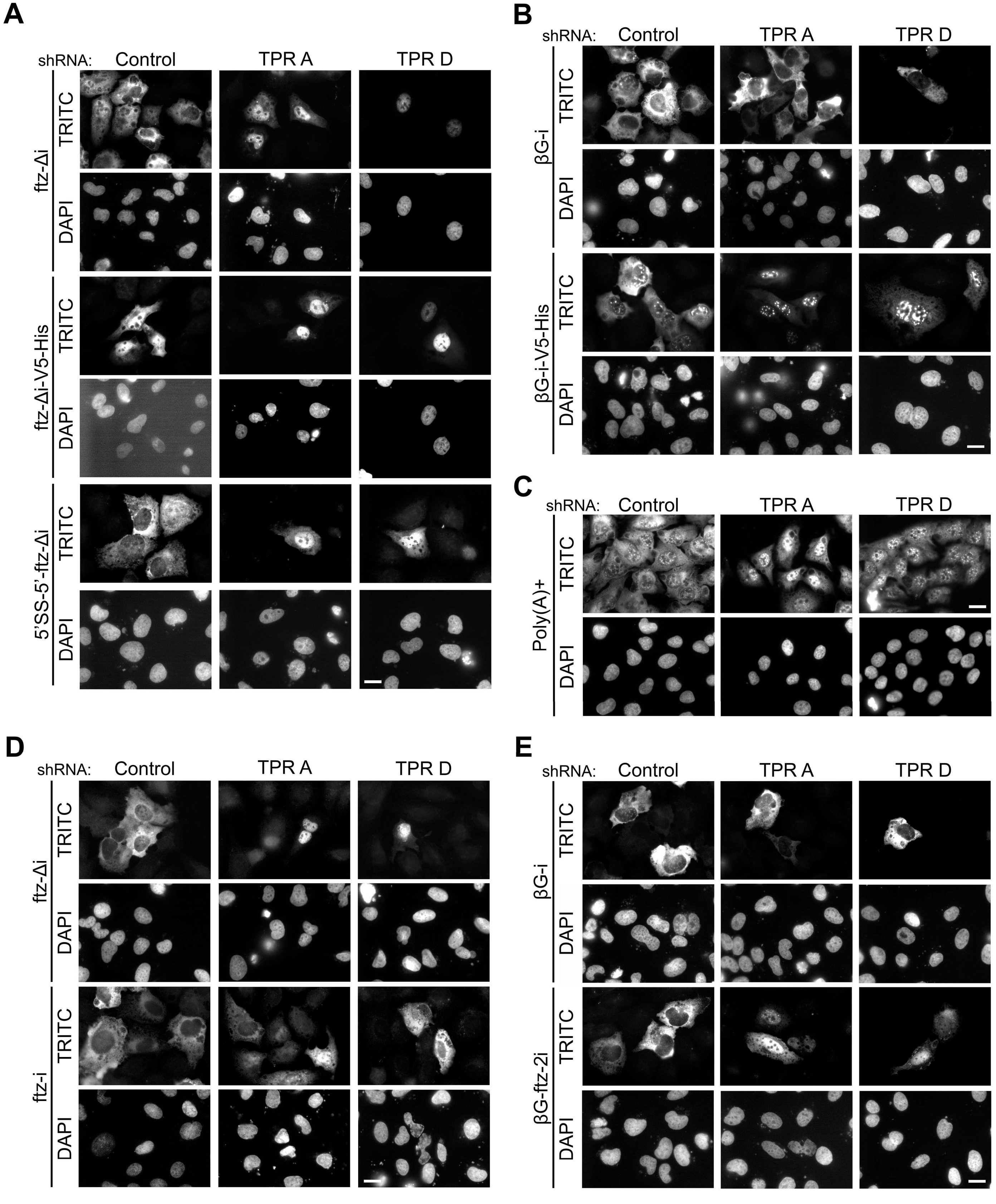

Figure S1. Reporter RNA localization with DAPI images

A) Related to Figure 1C, B) related to Figure 1F, C) related to Figure 1L, D) related to Figure 6B and E) related to Figure 6E. Note that each field of view has two images, RNA FISH (top) DAPI stain of DNA (bottom).

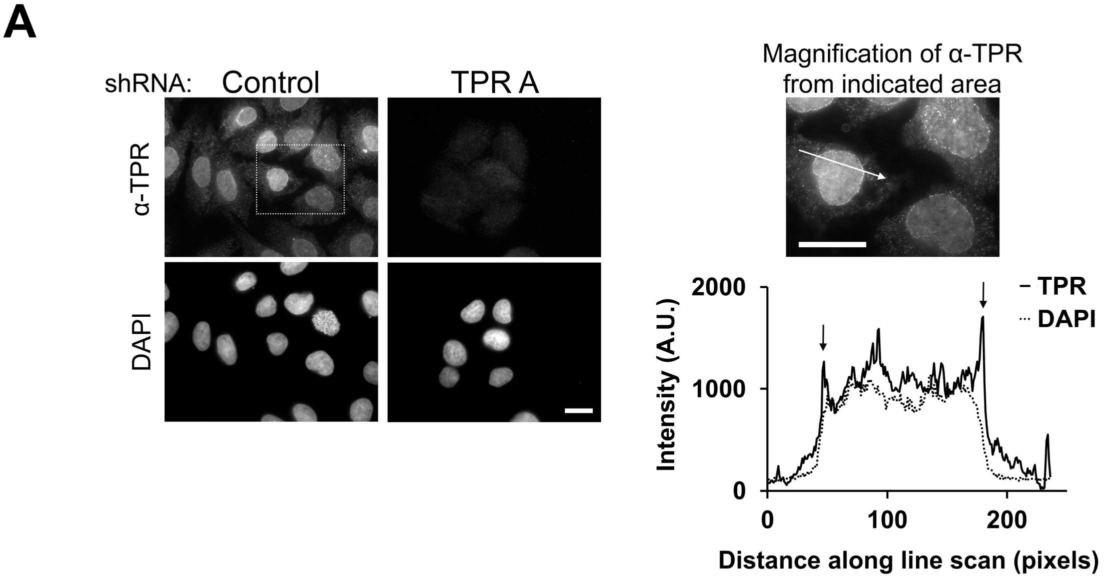
A
shRNA:
Control
TPR A
α-TPR
DAPI
Magnification of α-TPR from indicated area
TPR
DAPI
Intensity (A.U.)
2000
1000
0
0
100
200
Distance along line scan (pixels)

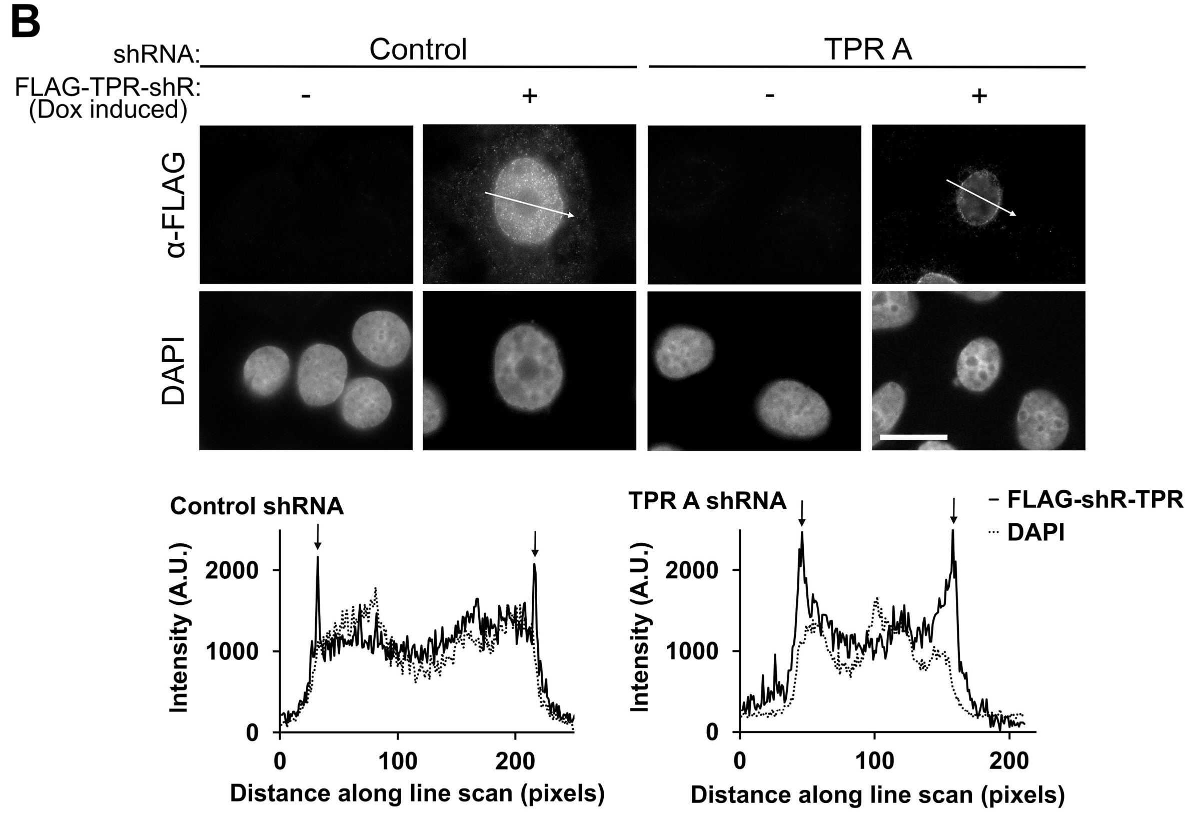
B
shRNA:
Control
TPR A
FLAG-TPR-shR:
(Dox induced)
-
+
-
+
α-FLAG
DAPI
Control shRNA
TPR A shRNA
FLAG-shR-TPR
DAPI
Intensity (A.U.)
2000
1000
0
0
100
200
Distance along line scan (pixels)

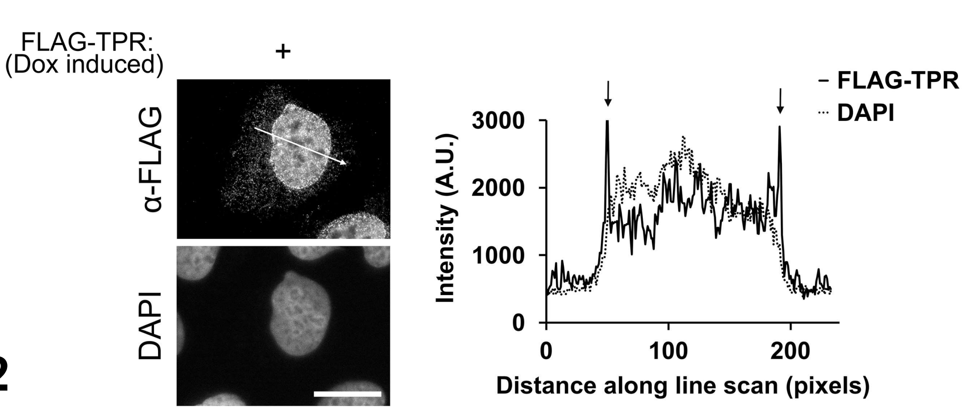
C
FLAG-TPR:
(Dox induced)
+
α-FLAG
DAPI
FLAG-TPR
DAPI
Intensity (A.U.)
3000
2000
1000
0
0
100
200
Distance along line scan (pixels)

Figure S2

Figure S2. (Related to Figure 1 and 2) Re-expression of shRNA resistant FLAG-TPR restores cytoplasmic accumulation of *ftz*-reporter RNA in TPR-depleted cells

A) Staining of endogenous TPR with specific mouse monoclonal antibodies against TPR (ab58344). Depletion of TPR by shRNA "TPR A" abolished all TPR staining indicating that TPR was successfully depleted in these cells. Shown inset is the magnification of TPR staining from indicated area in Control depleted cells; notice the characteristic nuclear rim staining of TPR. Scale bar = 10 μm. Linescan graph showing the intensity of TPR nuclear rim staining (compared to DAPI staining) along the white arrow. Note that each column is a single field of view with immunofluorescence (top) and DAPI staining of DNA (bottom).

B) Representative images showing expression of FLAG-TPR-shR in cells depleted by Control or TPR A shRNA in a U2OS Flp-In cell line. FLAG-TPR-shR localizes to the nuclear rim similar to endogenous TPR as shown in Figure S2A. FLAG-TPR-shR was visualized by immunofluorescence staining using FLAG-M2 antibody. Scale bar = 10 μm. Linescan graph showing that doxycycline-induced FLAG-TPR-shR localizes to the nuclear rim, similar to endogenous TPR. Note that each column is a single field of view with immunofluorescence (top) and DAPI staining of DNA (bottom).

C) Similar to (B), except that FLAG-TPR is re-expressed from a Flp-In U2OS cell line following doxycycline treatment, related to Figure 7D-F. Note that this is a single field of view with immunofluorescence (top) and DAPI staining of DNA (bottom).

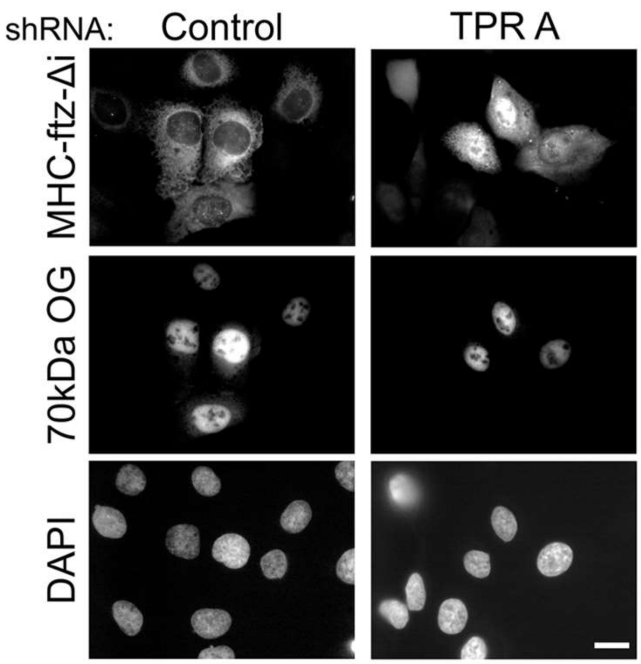

Figure S3 (Related to Figure 1J)

Representative images showing the localization of *MHC-ftz-Δi* reporter mRNA two hours post injection. *In vitro* synthesized RNA was microinjected into the nuclei of Control- or TPR- depleted cells, and incubated for 2 hours. Cells were then fixed and stained for *MHC-ftz-Δi* reporter RNA and DNA using DAPI. Each column represents one field of view with FISH against *MHC-ftz-Δi* mRNA (top row), 70kDa Oregon Green dextran ("70kDa OG") marker – to show the microinjected compartment (middle row) and PADI staining of DNA (bottom row). Scale bar = 10 μM.

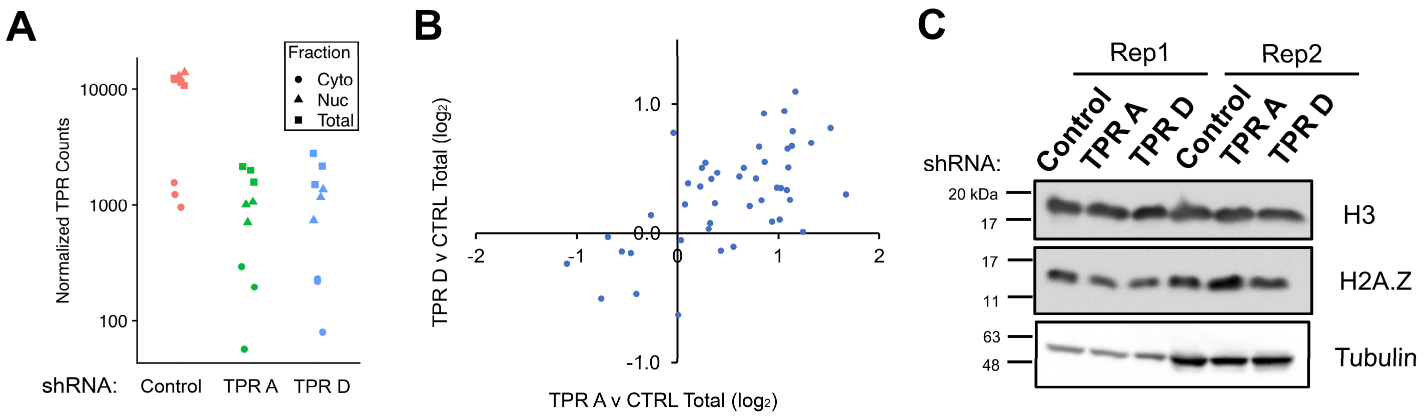

Figure S4. (Related to Figure 3) TPR-depletion induces the expression of histone mRNAs

A) Normalized counts from RNA-Seq showing that TPR was successfully depleted for either total RNA, cytoplasmic or nuclear fractions.

B) Normalized counts from RNA-Seq showing that TPR-depletion induces the expression of *histone* mRNAs.

C) Immunoblots of histone H3 or H2A.Z. from lysates collected from U2OS cells treated with control, “TPR A” or “TPR D” shRNA.

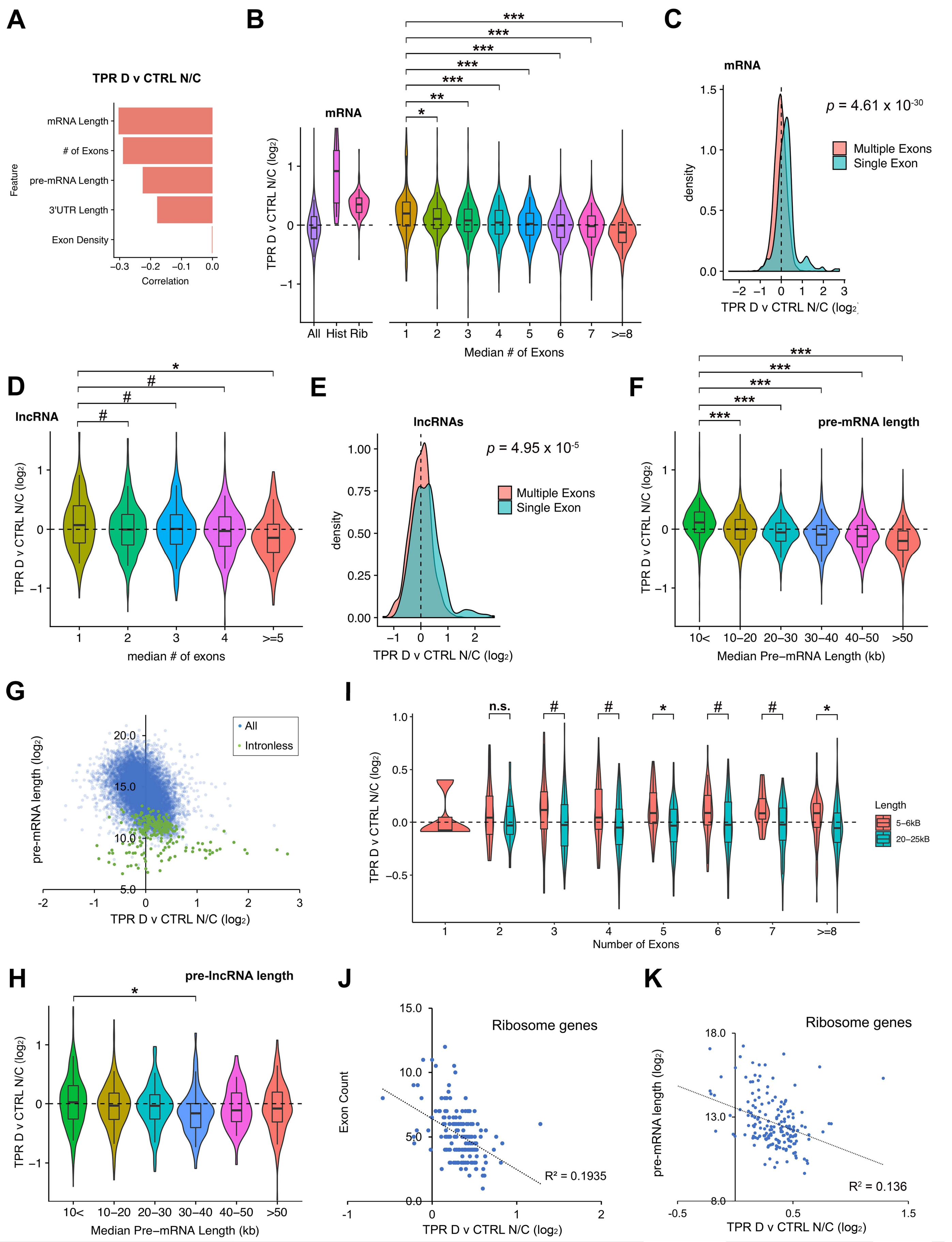
A
TPR D v CTRL N/C
mRNA Length
# of Exons
pre-mRNA Length
3'UTR Length
Exon Density
Feature
Correlation
B
mRNA
TPR D v CTRL N/C (log2)
All Hist Rib
Median # of Exons
C
mRNA
p = 4.61 x 10-30
Multiple Exons
Single Exon
density
TPR D v CTRL N/C (log2)
D
lncRNA
median # of exons
E
lncRNAs
p = 4.95 x 10-5
Multiple Exons
Single Exon
F
pre-mRNA length
Median Pre−mRNA Length (kb)
G
All
Intronless
pre-mRNA length (log2)
I
Length
5−6kB
20−25kB
Number of Exons
H
pre-lncRNA length
Median Pre−mRNA Length (kb)
J
Ribosome genes
Exon Count
R² = 0.1935
K
Ribosome genes
pre-mRNA length (log2)
R² = 0.136

Figure S5

Figure S5. (Related to Figure 4) TPR-depletion inhibits the nuclear export of mRNAs and lncRNAs transcribed from short pre-mRNAs, including intronless and intron-poor genes

A-K) Same as in Figure 4, except that the “TPR D” shRNA was used.

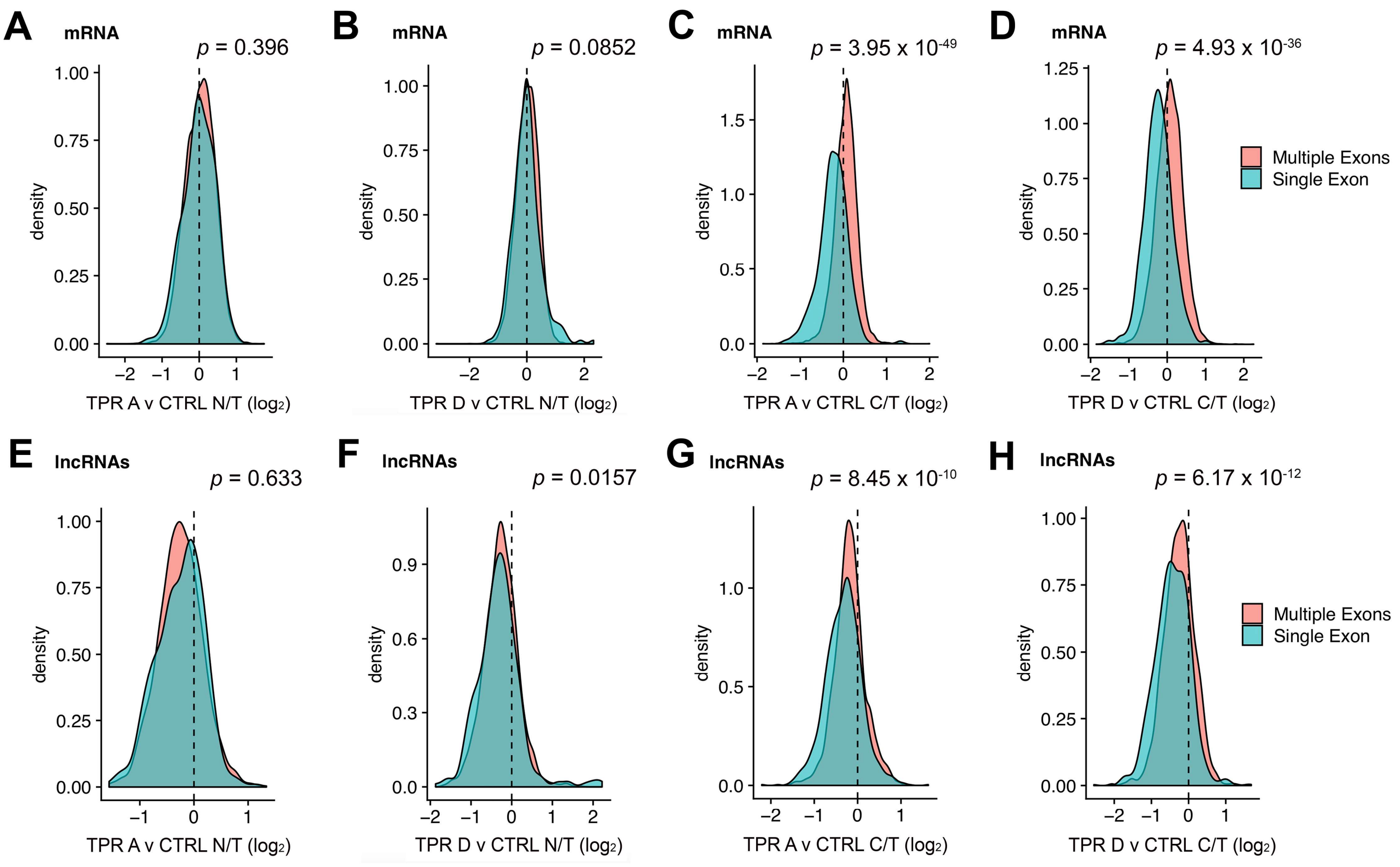

Figure S6. (Related to Figure 4 and S5)

A-D) Related to Figure 4C and S5C. Density plot showing intronless and multi-exon mRNAs distribution compared to nuclear/total (N/T) or cytoplasmic/total (C/T) fold change upon TPR depletion for “TPR A” or “TPR D” shRNA. Mann-Whitney-Wilcoxon statistical tests was performed.

E-H) Related to Figure 4E and S5E. Density plot showing that intronless and multi-exon lncRNAs distribution compared to N/T or C/T fold change upon TPR depletion for “TPR A” or “TPR D” shRNA.

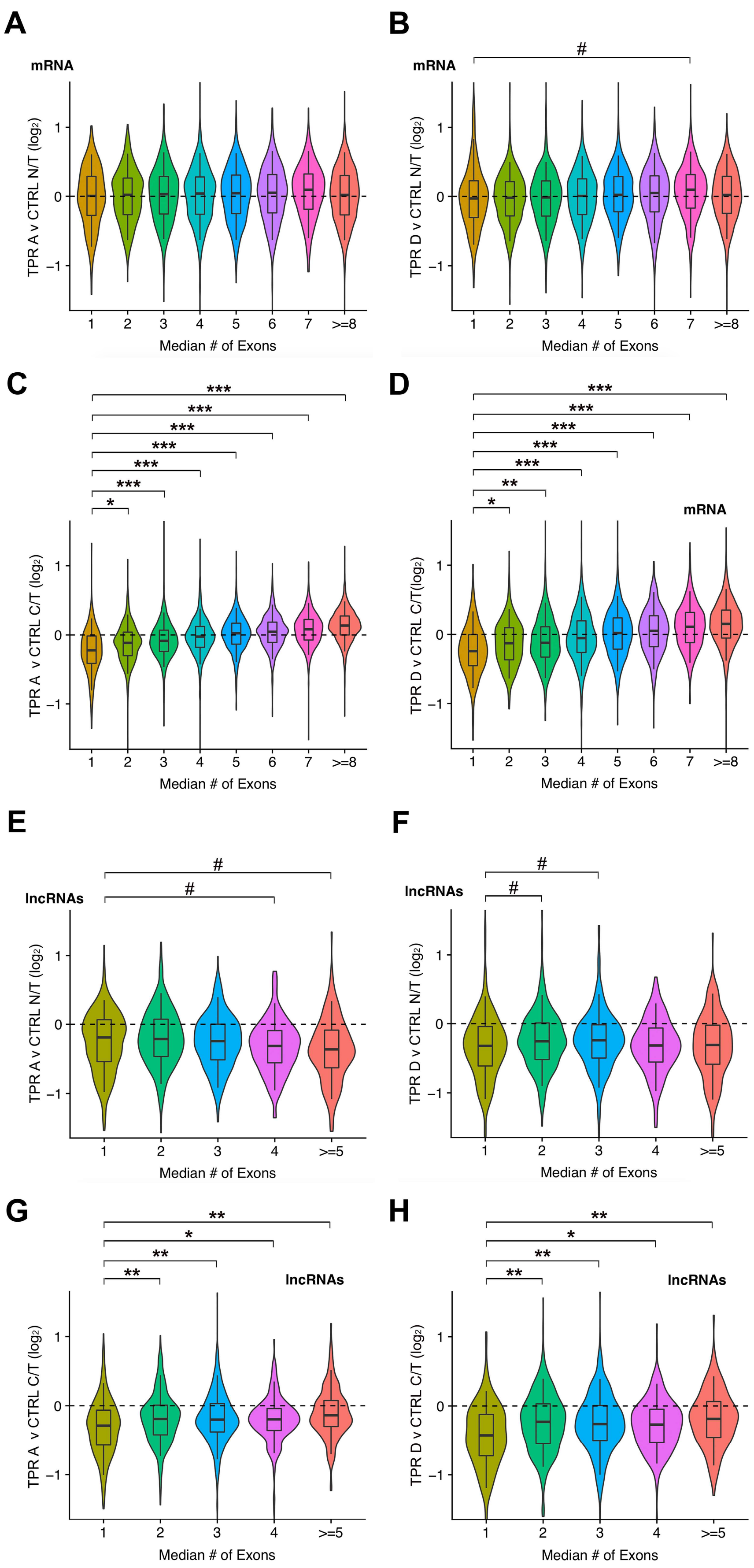
A
mRNA
TPR A v CTRL N/T (log2)
Median # of Exons
B
mRNA
#
TPR D v CTRL N/T (log2)
Median # of Exons
C
TPR A v CTRL C/T (log2)
Median # of Exons
D
mRNA
TPR D v CTRL C/T(log2)
Median # of Exons
E
lncRNAs
TPR A v CTRL N/T (log2)
Median # of Exons
F
lncRNAs
TPR D v CTRL N/T (log2)
Median # of Exons
G
lncRNAs
TPR A v CTRL C/T (log2)
Median # of Exons
H
lncRNAs
TPR D v CTRL C/T (log2)
Median # of Exons

Figure S7

Figure S7. (Related to Figure 4 and S5)

A-D) Related to Figure 4B and S5B. Violin plot of N/T or C/T fold change upon TPR depletion for mRNAs with various number of exons. Note that mRNAs with more exons are more cytoplasmic upon TPR depletion. Mann-Whitney-Wilcoxon statistical tests with Bejamini-Hochberg adjustment for multiple comparisons was performed, $^{\#}$ $p < 0.05$, * $p < 10^{-3}$, ** $p < 10^{-5}$ and *** $p < 10^{-10}$.

E-H) Related to Figure 4D and S5D. Violin plot of N/T or C/T fold change upon TPR depletion for lncRNAs with various number of exons. LncRNAs with more exons are more cytoplasmic upon TPR depletion.

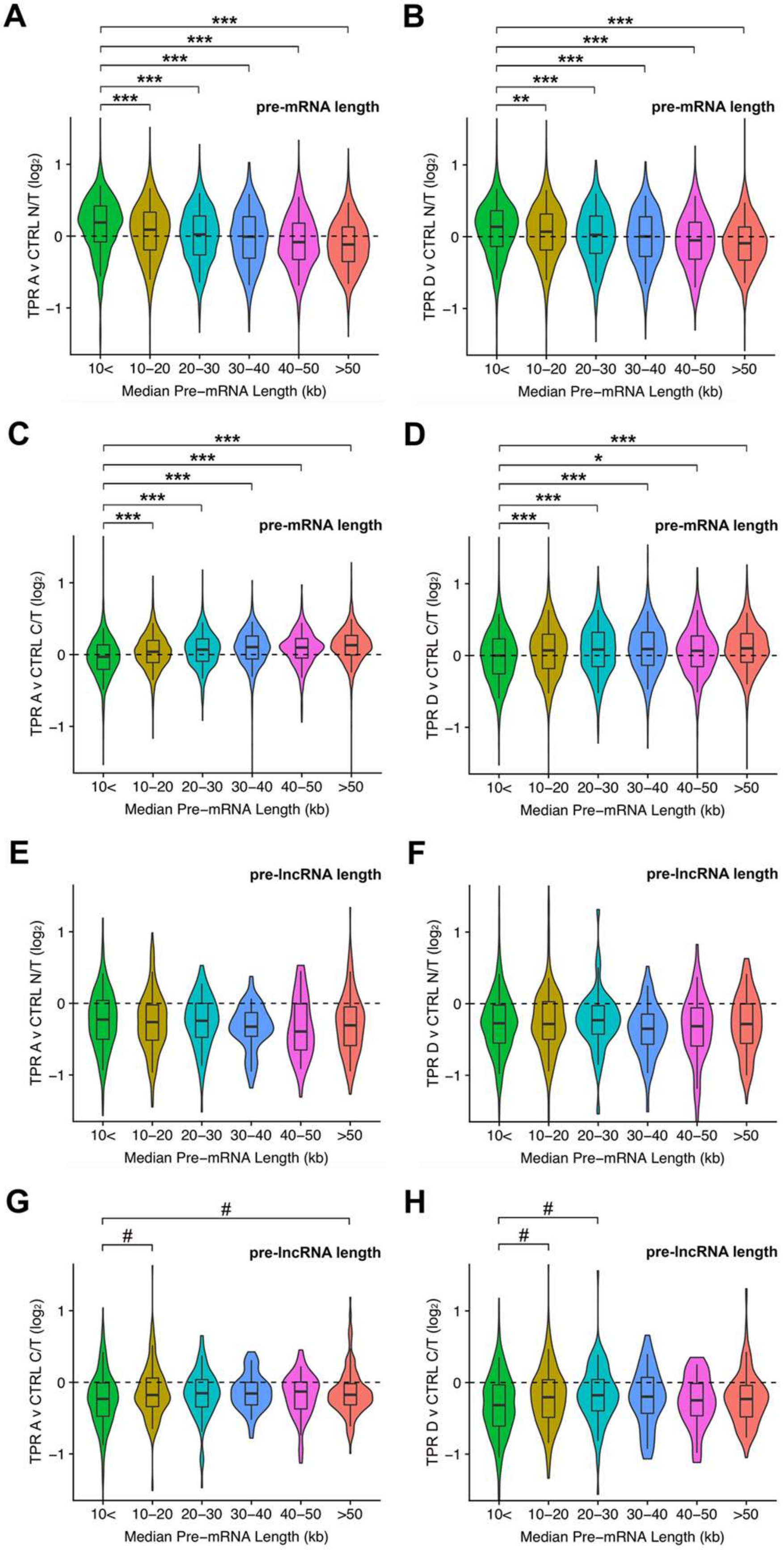
A
B
C
D
E
F
G
H
pre-mRNA length
pre-lncRNA length
TPR A v CTRL N/T (log2)
TPR D v CTRL N/T (log2)
TPR A v CTRL C/T (log2)
TPR D v CTRL C/T (log2)
10< 10−20 20−30 30−40 40−50 >50
Median Pre−mRNA Length (kb)

Figure S8

Figure S8. (Related to Figure 4 and S5)

A-D) Related to Figure 4F and S5F. Violin plot of N/T or C/T fold change upon TPR depletion for pre-mRNAs of varying lengths in 10 kb windows. Note that pre-mRNAs of increasing lengths are more cytoplasmic upon TPR depletion and shorter pre-mRNAs are more nuclear upon TPR depletion. Mann-Whitney-Wilcoxon statistical tests with Bejamini-Hochberg adjustment for multiple comparisons was performed, $^{\#}$ $p < 0.05$, * $p < 10^{-3}$, ** $p < 10^{-5}$ and *** $p < 10^{-10}$.

E-H) Related to Figure 4H and S5H. Violin plot of N/T or C/T fold change upon TPR depletion for pre-lncRNAs of varying lengths.

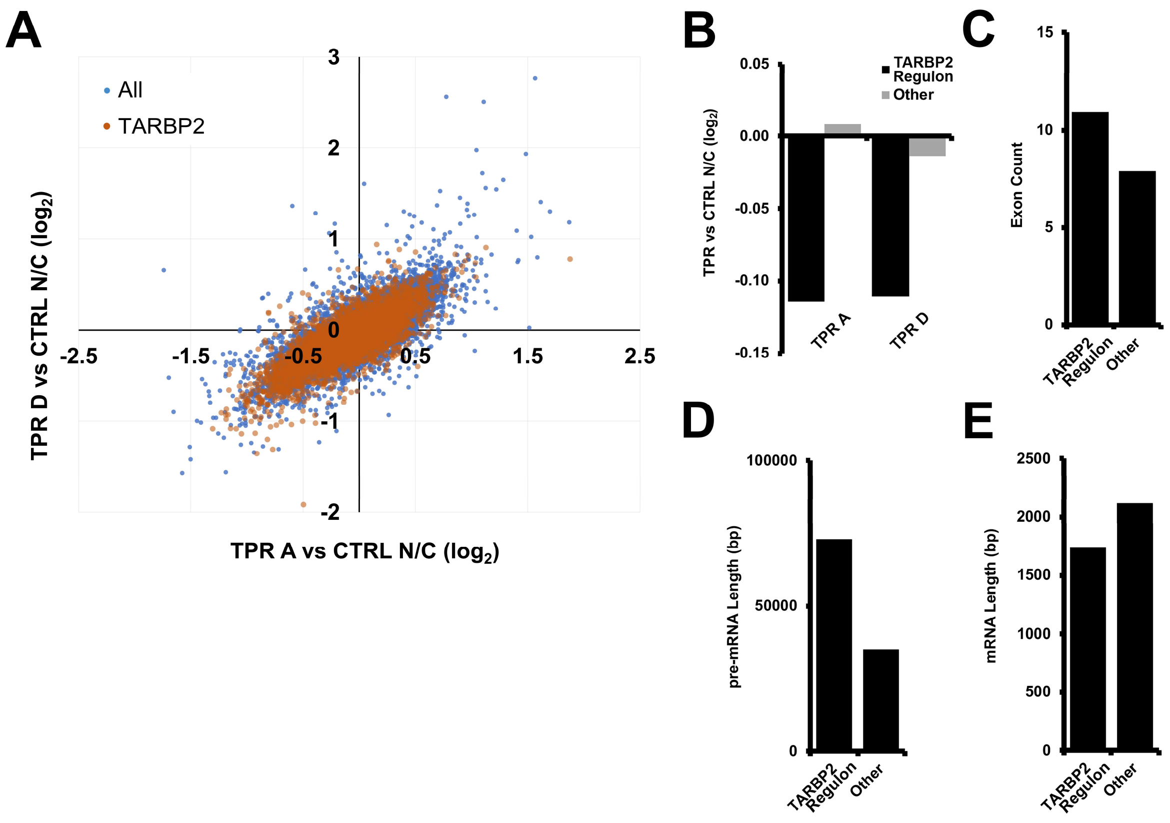

Figure S9. TPR-depletion affect the nuclear retention of "TARBP2 regulons"

A) "TABP2 regulons" are slightly more cytoplasmic in TPR depleted cells (compare orange dots with blue). "TARBP2 regulons" are derived from TARBP2 iCLIP dataset (Fish et al 2019).

B) Average fold change (TPR v CTRL N/C) for "TARBP2 regulons" are cytoplasmic compared to the rest of the dataset ("other").

C-E) An analysis of "TARBP2 regulon" mRNAs. These have more exons (C) and have longer pre-mRNA (D) than other mRNAs. The average exon length of these mRNAs is similar to that of the entire dataset (E).
